# Can quality improvement improve the quality of care? A systematic review of reported effects and methodological rigor in plan-do-study-act projects

**DOI:** 10.1186/s12913-019-4482-6

**Published:** 2019-10-04

**Authors:** Søren Valgreen Knudsen, Henrik Vitus Bering Laursen, Søren Paaske Johnsen, Paul Daniel Bartels, Lars Holger Ehlers, Jan Mainz

**Affiliations:** 1 Danish Center for Clinical Health Services Research (DACS), Department of Clinical Medicine, Aalborg University, Mølleparkvej 10, 9000 Aalborg, Denmark; 2 Psychiatry, Aalborg University Hospital, The North Denmark Region Mølleparkvej 10, 9000 Aalborg, Denmark; 3 Danish Center for Healthcare Improvements (DCHI), Aalborg University, Fibigerstræde 11, 9220 Aalborg Øst, Denmark; 4 Danish Clinical Registries, Denmark, Nrd. Fasanvej 57, 2000 Frederiksberg, Denmark; 5 Department for Community Mental Health, Haifa University, Haifa, Israel; 6 Department of Health Economics, University of Southern Denmark, Odense, Denmark

**Keywords:** PDSA, Plan-do-study-act, Quality, Health services research, Quality improvement

## Abstract

**Background:**

The Plan-Do-Study-Act (PDSA) method is widely used in quality improvement (QI) strategies. However, previous studies have indicated that methodological problems are frequent in PDSA-based QI projects. Furthermore, it has been difficult to establish an association between the use of PDSA and improvements in clinical practices and patient outcomes. The aim of this systematic review was to examine whether recently published PDSA-based QI projects show self-reported effects and are conducted according to key features of the method.

**Methods:**

A systematic literature search was performed in the PubMed, Embase and CINAHL databases. QI projects using PDSA published in peer-reviewed journals in 2015 and 2016 were included. Projects were assessed to determine the reported effects and the use of the following key methodological features; iterative cyclic method, continuous data collection, small-scale testing and use of a theoretical rationale.

**Results:**

Of the 120 QI projects included, almost all reported improvement (98%). However, only 32 (27%) described a specific, quantitative aim and reached it. A total of 72 projects (60%) documented PDSA cycles sufficiently for inclusion in a full analysis of key features. Of these only three (4%) adhered to all four key methodological features.

**Conclusion:**

Even though a majority of the QI projects reported improvements, the widespread challenges with low adherence to key methodological features in the individual projects pose a challenge for the legitimacy of PDSA-based QI. This review indicates that there is a continued need for improvement in quality improvement methodology.

**Electronic supplementary material:**

The online version of this article (10.1186/s12913-019-4482-6) contains supplementary material, which is available to authorized users.

## Background

Plan-Do-Study-Act (PDSA) cycles are widely used for quality improvement (QI) in most healthcare systems where tools and models inspired by industrial management have become influential [1]. The essence of the PDSA cycle is to structure the process of improvement in accordance with the scientific method of experimental learning [2–5]. It is used with consecutive iterations of the cycle constituting a framework for continuous learning through testing of changes [6–10].

**Fig. 1:**
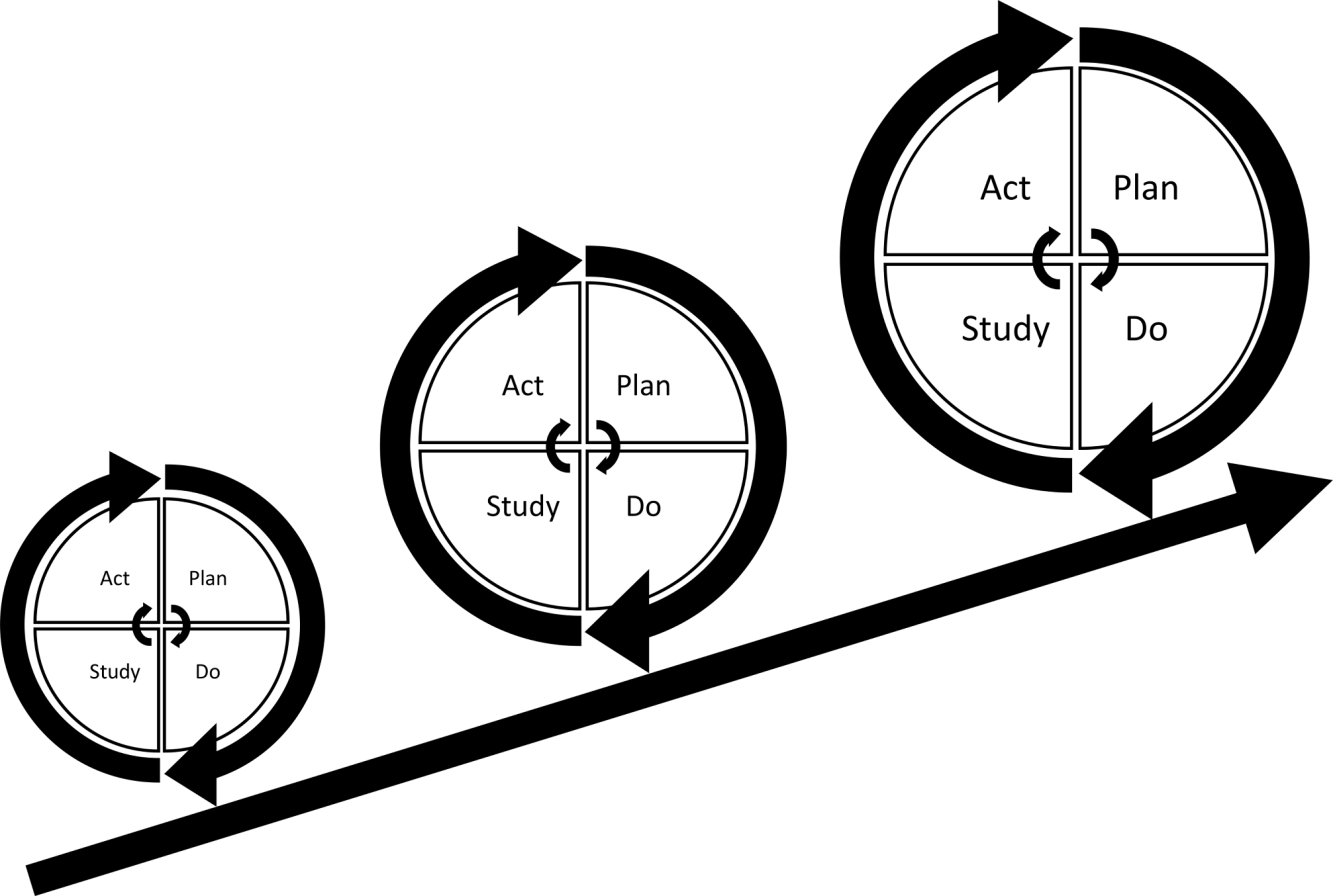
Plan-Do-Study-Act (PDSA) based quality improvement. Each cycle informs the subsequent cycle. Ideally, the complexity and size of the intervention is upscaled iteratively as time pass, knowledge is gained and quality of care is improved

The concept of improvement through iterative cycles has formed the basis for numerous structured QI approaches including Total Quality Management, Continuous Quality Improvement, Lean, Six Sigma and the Model for Improvement [4, 6, 10]. These “PDSA models” have different approaches but essentially consist of improvement cycles as the cornerstone combined with a bundle of features from the management literature. Especially within healthcare, several PDSA models have been proposed for QI adding other methodological features to the basic principles of iterative PDSA cycles. Key methodological features include the use of continuous data collection [2, 6, 8–13], small-scale testing [6, 8, 10, 11, 14–16] and use of a theoretical rationale [5, 9, 17–22]. Most projects are initiated in the complex social context of daily clinical work [12, 23]. In these settings, focus on use of these key methodological features ensures quality and consistency by supporting adaptation of the project to the specific context and minimizing the risk of introducing harmful or wasteful unintended consequences [10]. Thus, the PDSA cycle is not sufficient as a standalone method [4] and integration of the full bundle of key features is often simply referred to as the PDSA method (Fig. 1).

Since its introduction to healthcare in the 1990s, numerous QI projects have been based on the PDSA method [10, 24]. However, the scientific literature indicates that the evidence for effect is limited [10, 25–30]. The majority of the published PDSA projects have been hampered with severe design limitations, insufficient data analysis and incomplete reporting [12, 31]. A 2013 systematic review revealed that only 2/73 projects reporting use of the PDSA cycle applied the PDSA method in accordance with the methodological recommendations [10]. These methodological limitations have led to an increased awareness of the need for more methodological rigor when conducting and reporting PDSA-based projects [4, 10]. This challenge is addressed by the emergent field of Improvement Science (IS) which attempts to systematically examine methods and factors that best facilitate QI by drawing on a range of academic disciplines and encourage rigorous use of scientific methods [5, 12, 32, 33]. It is important to make a distinction between local QI projects, where the primary goal is to secure a change, and IS, where the primary goal is directed at evaluation and scientific advancement [12].

In order to improve local QI projects, Standards for Quality Improvement Reporting Excellence (SQUIRE) guidelines have been developed to provide a framework for reporting QI projects [18, 34]. Still, it remains unclear to what extent the increasing methodological awareness is reflected in PDSA-based QI projects published in recent years. Therefore, we performed a systematic review of recent peer-reviewed publications reporting QI projects using the PDSA methodology in healthcare and focused on the use of key features in the design and on the reported effects of the projects.

## Methods

The key features of PDSA-based QI projects were identified, and a simple but comprehensive framework was constructed. The review was conducted in adherence with the Preferred Reporting Items for Systematic Reviews and Meta-Analyses (PRISMA) statement [35].

### The framework

Informed by recommendations for key features in use and support of PDSA from literature specific to QI in healthcare the following key features were identified:

- Use of an iterative cyclic method [6–10]
- Use of continuous data collection [2, 6, 8–13]
- Small-scale testing [6, 8, 10, 11, 14–16]
- Explicit description of the theoretical rationale of the projects [5, 9, 17–22]

Aiming for conceptual simplicity, we established basic minimum requirements for the presence of the key features operationalizing them into binary (yes/no) variables. General characteristics and supplementary data that elaborated the use of the key features were operationalized and registered as categorical variables. See Table 1 for an overview of the framework and Additional file 1 for a more in-depth elaboration of the definitions used for the key features. Since a theoretical rationale can take multiple forms, the definition for this feature was taken from the recent version of the SQUIRE guidelines [18].

**Table 1:** Framework based on key features of data-driven PDSA projects

Feature	Description of feature	Criteria for key feature	Supplementary features
Documentation	Sufficient documentation of PDSA cycles is set as a requirement for the project to be analysed against the full framework	Individual cycles being described, with or without details on stages within cycles	
Iterative cycles	The iterative approach essentially is the linking of knowledge gained from one PDSA cycle to the next. Through multiple cycles knowledge is built and interventions are either adopted, adapted or abandoned.	At least two successive cycles, linked by theme and function, in which lessons from one cycle informed the next	- Nature of cycles - Several tests of change in a cycle
Small-scale testing	Small tests of change allow unexpected obstacles and unforeseen effects to be caught, and trust in the project to be built before full-scale implementation.	The change(s) were introduced on a scale smaller than an entire department/treatment unit tested, before a full-scale test was begun	- Scope of QI effort - Pre-project intention of testing under different conditions - Type of scaling when using small scale
Continuous data collection	Using continuous data collection is necessary to understand the inherent variation within the system and determine whether the process is stable.	Data was collected regularly over time, with three or more consecutive data points	- Main type of data used - Measurement type - Use of baseline - Type of time series diagram
Theoretical rationale	Improvers always use theories when developing and executing their projects, but stating them can help both in designing, executing and especially evaluating it, and helps in articulation of assumptions and predictions of why the project will result in improvement in their context	Informal or formal frameworks, models, concepts and/or theories used to explain the problem, any reasons or assumptions that were used to develop the project(s) and reasons why the project(s) was expected to work	- Evidence based inspiration for the need for improvement - Origin of inspiration for QI intervention

Since no formal standardized requirements for reporting PDSA-based QI projects across journals are established, not all report the individual PDSA cycles in detail. To ensure that variation in use of key features were inherent in the conduct of the projects and not just due to differences in the reporting, sufficient documentation of PDSA cycles was set as a requirement for analysis against the full framework.

#### Self-reported effects

A pre-specified, quantitative aim can assist to facilitate evaluation of whether the changes represent clinically relevant improvements when using the PDSA method [16]. Self-reported effects of the projects were registered using four categories: 1) Quantitative aim set and reached; 2) No quantitative aim set, improvement registered; 3) Quantitative aim set but not reached; 4) No quantitative aim and no improvement registered.

### Systematic review of the literature

The target of the literature search was peer-reviewed publications that applied the PDSA cycle as the main method for a QI project in a healthcare setting. The search consisted of the terms ([*‘PDSA’* OR ‘*plan-do-study-act’*] AND [‘*quality*’ OR ‘*improvement*’]). The terms were searched for in title and abstract. No relevant MeSH terms were available. To get a contemporary status of the QI field, the search was limited to QI projects published in 2015 and 2016. PubMed, Embase and CINAHL databases were searched with the last search date being 2nd of March 2017.

#### Study selection

The following inclusion criteria were used: Peer-reviewed publications reporting QI projects using the PDSA methodology in healthcare, published in English. Exclusion criteria were: IS studies, editorials, conference abstracts, opinions and audit articles, reviews or projects solely involving teaching the PDSA method.

Two reviewers (SVK and HVBL) performed the screening process independently. Title and abstract were screened for inclusion followed by an assessment of the full text according to the eligibility criteria. This was performed in a standardized manner with the Covidence software. Disagreements were resolved by consensus.

#### Data collection process

A data collection sheet was developed and pilot tested. The subsequent refinement resulted in a standardized sheet into which data were extracted independently by SVK and HVBL.

#### Data items

Data from the key and supplementary features were extracted in accordance with the framework. The binary data were used to grade QI projects on a scale of 0–4, based on how many of the four key features were applied. Data were analyzed in STATA (version 15.0, StataCorp LLC).

## Results

### Study selection

#### Selection process

**Fig. 2:**
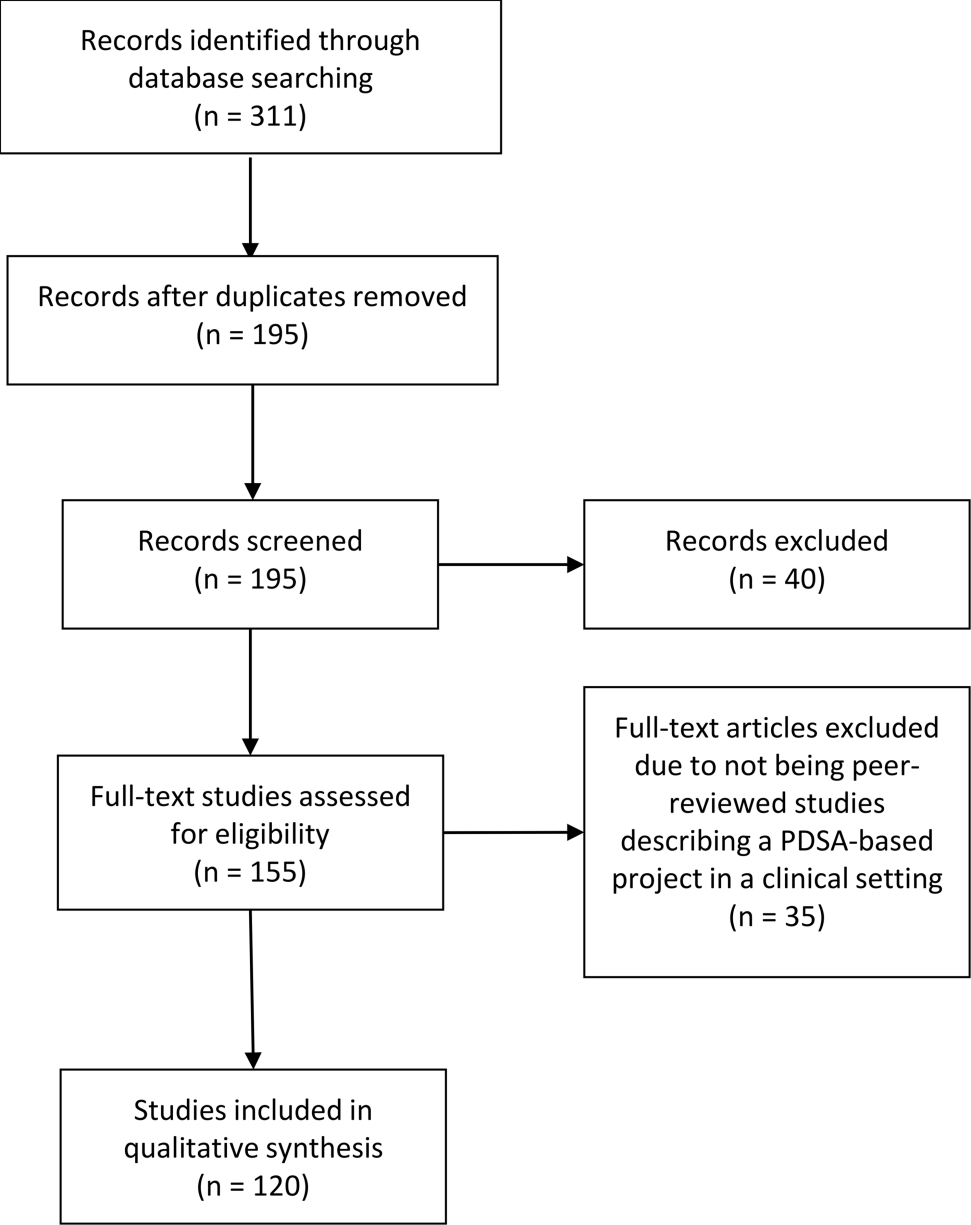
PRISMA diagram

The search identified 311 QI projects of which 195 remained after duplicate removal. A total of 40 and 35 projects were discarded after screening abstracts and full texts, respectively. Hence, a total of 120 projects met the inclusion criteria and were included in the review (see Fig. 2).

An overview of general characteristics, supplementary features and self-reported effects of the included projects are presented in Table 2.

**Table 2:** Overview of self-reported effects, general characteristics and supplementary features of the included projects

Self-reported effects of QI project		27%	32/120	Quantitative aim was achieved
		57%	68/120	Positive change - no quantitative aim
		15%	18/120	Positive change - quantitative aim not reached
		2%	2/120	No quantitative aim and no improvement
Included projects (*n* = 120)				
General characteristics	Journal	44%	53/120	BMJ Quality Improvement Reports
		5%	6/120	Pediatrics
		4%	5/120	Journal of Oncology Practice
		47%	56/120	Other journals
	Country	43%	52/120	USA
		36%	43/120	The UK
		5%	6/120	Canada
		4%	5/120	Singapore
		3%	4/120	Saudi Arabia
		2%	2/120	Australia
		7%	8/120	Other
	Reach	86%	103/120	Local
		11%	13/120	Regional
		3%	3/120	Nationwide
		1%	1/120	Not stated
	Area of healthcare	57%	68/120	Department
		30%	36/120	Hospital-wide
		13%	16/120	Other
	Department specialty	30%	28/94	Pediatrics
		14%	13/94	ICU/ED
		13%	12/94	Surgery
		12%	11/94	Psychiatry
		11%	10/94	Internal Medicine
		21%	20/94	Other
	Supporting framework	58%	70/120	Not stated
		33%	40/120	Model for Improvement
		9%	11/120	Lean, Six-sigma or other frameworks
Documentation of PDSA cycles	Documentation category	19%	23/120	No details of cycles
		21%	25/120	Themes of cycles but no additional details
		50%	60/120	Details of individual cycles but not stages of cycles
		10%	12/120	Details of cycles including separate information on stages of cycles
Included projects (*n* = 72), 48 excluded due to lack of documentation criteria				
Iterative approach characteristics	Nature of cycles	3%	2/72	Single isolated cycle
		18%	13/72	Multiple isolated cycles
		57%	41/72	Iterative chain
		5%	4/72	Multiple chains of isolated cycles
		17%	12/72	Mix of iterative chains and isolated cycles
	Several tests of change in a cycle	76%	55/72	Yes
		24%	17/72	No
Small scale testing characteristics	Scope of QI effort	40%	29/72	Testing
		46%	33/72	Implementing
		0%	0/72	Spreading
		13%	9/72	Testing and implementing
		1%	1/72	Testing, implementing and spreading
	Pre-project intention of testing under different conditions	0%	0/72	Yes
		100%	72/72	No
	Type of scaling when using small scale	10%	1/10	Unclear
		70%	7/10	Increasing
		20%	2/10	Non-increasing
Continuous data collection characteristics	Main type of data used	72%	52/72	Quantitative data
		22%	16/72	Quantitative data with supplementary qualitative data
		4%	3/72	Quantitative & qualitative data
		1%	1/72	Quantitative data but not presented
	Measurement type	67%	48/72	Regular three or more data points
		25%	18/72	Before and after or per PDSA cycle(s)
		7%	5/72	Single data point after PDSA cycle(s)
		1%	1/72	No quantitative data reported
	Use of baseline	90%	65/72	Yes
		10%	7/72	No
	Type of time series diagram	50%	24/48	Run Chart
		50%	24/48	Control Chart
Theoretical rationale characteristics	Evidence based inspiration for the need for improvement	94%	68/72	Yes
		6%	4/72	No
	Origin of inspiration for QI intervention	36%	26/72	External knowledge, scientific literature, previous QI or benchmarking
		29%	21/72	Internally developed knowledge, logical thinking
		14%	10/72	A combination of internal and external
		21%	15/72	Not stated

### General characteristics

#### Country and journal

The included QI projects originated from 18 different countries including the USA (*n* = 52), the *UK* (*n* = 43), Canada (*n* = 6), Singapore (*n* = 5), Saudi Arabia (*n* = 4), Australia (*n* = 2) and one each from eight other countries. Fifty different journals had published QI projects with the vast majority (*n* = 53) being from BMJ Quality Improvement Reports. See Additional file 2 for a full summery of the findings.

#### Area and specialty

In terms of reach, most were local (*n* = 103) followed by regional (*n* = 13) and nationwide (*n* = 3). The areas of healthcare were primarily at departmental (*n* = 68) and hospital level (*n* = 36). Many different specialties were represented, the most common being pediatrics (*n* = 28), intensive or emergency care (*n* = 13), surgery (*n* = 12), psychiatry (*n* = 11) and internal medicine (*n* = 10).

#### Supporting framework

Most QI projects did not state using a supporting framework (*n* = 70). However, when stated, most used The Model for Improvement (*n* = 40). The last (*n* = 10) used Lean, Six-sigma or other frameworks.

### Reported effects

All 120 projects included were assessed for the self-reported effects. Overall, 118/120 (98%) projects reported improvement. Thirty-two (27%) achieved a pre-specified aim set in the planning process, whereas 68 (57%) reported an improvement without a pre-specified aim. Eighteen projects (15%) reported setting an aim and not reaching it while two (2%) projects did not report a pre-specified aim and did not report any improvement.

### Documentation

Seventy-two projects had sufficient documentation of the PDSA cycles. Sixty of these contained information on individual stages of cycles, while 12 in addition presented detailed information on the four stages of the PDSA cycles.

### Application of key features of PDSA

**Fig. 3:**
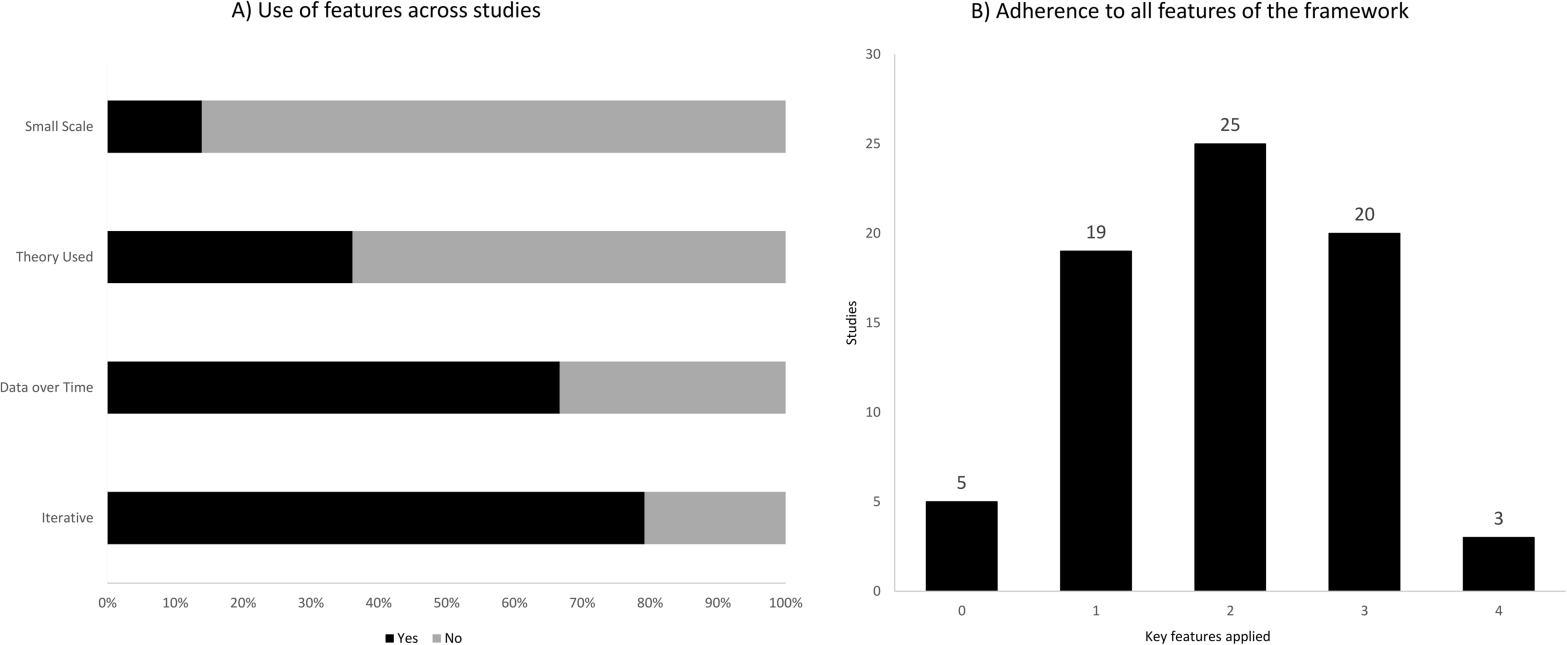
**a**) Bar-chart depicting how often the four key features were used across the projects. **b**) Bar-chart depicting the number of projects, which had used zero to four key features

The application of the key PDSA features appeared to be highly inconsistent. The iterative method was used in 75 projects (79%), continuous data collection in 48 (67%), an explicit theoretical rational was present in 26 (36%) projects and small-scale testing was carried out by 10 (14%) (Fig. 3a). All key features of the method were applied in 3/72 projects (4%), while 20 (28%), 26 (36%), and 18 (25%) used three, two, and one feature respectively. Five projects (7%) lacked all features (Fig. 3b). See Additional file 3 for a full summary of the findings.

#### Iterative cycles

Fifty-seven projects (79%) had a sequence of cycles where one informed the actions of the next. A single iterative chain of cycles was used in 41 (57%), while four (5%) had multiple isolated iterative chains and 12 (17%) had a mix of iterative chains and isolated cycles. Of the 15 projects using non-iterative cycles, two reported a single cycle while 13 used multiple isolated cycles. The majority (55/72) (76%) tested one change per cycle.

#### Small scale testing

The testing of changes in a small scale was carried out by 10 projects (14%), of which seven did so in an increasing scale, while two kept testing at the same scale. It was unclear which type of scaling was used in the remaining project. Sixty-two projects (86%) carried out testing on an entire department or engaged in full-scale implementation before having tested the improvement intervention.

#### Continuous data collection

Continuous measurements over time with three or more data points at regular intervals were used by 48 (67%) out of 72 projects. Of these 48, half used run charts, while the other half used control charts. Other types of data measurement such as before and after or per PDSA cycle or having a single data point as outcome after cycle(s) was done by 18 (25%) and 5 (7%), respectively. One project did not report their data. Sixty-five projects (90%) used a baseline measurement for comparison.

#### Theoretical rationale

Twenty-six (36%) out of 72 projects explicitly stated the theoretical rationale of the project describing why it was predicted to lead to improvement in their specific clinical context. In terms of inspiration for the need for improvement 68 projects (94%) referred to scientific literature. For the QI interventions used in the projects 26 (36%) found inspiration in externally existing knowledge in forms of scientific literature, previous QI projects or benchmarking. Twenty-one (29%) developed the projects themselves, 10 (14%) used existing knowledge in combination with own ideas while 15 (21%) did not state the source.

## Discussion

In this systematic review nearly all PDSA-based QI projects reported improvements. However, only approximately one out of four projects had defined a specific quantitative aim and reached it. In addition, only a small minority of the projects reported to have adhered to all four key features recommended in the literature to ensure the quality and adaptability of a QI project.

The claim that PDSA leads to improvement should be interpreted with caution. The methodological limitations in many of the projects makes it difficult to draw firm conclusions about the size and the causality of the reported improvements in quality of care. The methodological limitations question the legitimacy of PDSA as an effective improvement method in health care. The widespread lack of theoretical rationale and continuous data collection in the projects makes it difficult to track and correct the process as well as to relate an improvement to the use of the method [10, 11]. The apparent limited use of the iterative approach and small-scale-testing constitute an additional methodological limitation. Without these tools of testing and adapting one can risk introducing unintended consequences [1, 36]. Hence, QI initiatives may potentially tamper with the system in unforeseen ways creating more harm and waste than improvement. The low use of small-scale-testing could perhaps originate in a widespread misunderstanding that one should test large-scale to get a proper statistical power. However, this is not necessarily the case with PDSA [15].

There is no simple answer to this lack of adherence to the key methodological features. Some scholars claim that even though the concept of PDSA is relatively simple it is difficult to master in reality [4]. Some explanations to this have been offered including an urge to favour action over evidence [36], an inherent messiness in the actual use of the method [11], its inability to address “big and hairy” problems [37], an oversimplification of the method, and an underestimation of the required resources and support needed to conduct a PDSA-based project [4].

In some cases, it seems reasonable that the lack of adherence to the methodological recommendations is a problem with documentation rather than methodological rigor, e.g. the frequent lack of small-scale pilot testing may be due to the authors considering the information too irrelevant, while still having performed it in the projects.

Regarding our framework one could argue that it has too many or too few key features to encompass the PDSA method. The same can be said about the supplementary features where additional features could also have been assessed e.g. the use of Specific, Measurable, Attainable, Relevant and Timebound (SMART) goals [14]. It has been important for us to operationalize the key features so their presence easily and accurately can be identified. Simplification carries the risk of loss of information but can be outweighed by a clear and applicable framework.

This review has some limitations. We only included PDSA projects reported in peer-reviewed journals, which represents just a fraction of all QI projects being conducted around the globe. Further, it might be difficult to publish projects that do not document improvements. This may introduce potential publication bias. Future studies could use the framework to examine the grey literature of evaluation reports etc. to see if the pattern of methodological limitations is consistent. The fact that a majority of the projects reported positive change could also indicate a potential bias. For busy QI practitioners the process of translating a clinical project into a publication could well be motivated by a positive finding with projects with negative effects not being reported. However, we should not forget that negative outcome of a PDSA project may still contribute with valuable learning and competence building [4, 6].

The field of IS and collaboration between practitioners and scholars has the potential to deliver crucial insight into the complex process of QI, including the difficulties with replicating projects with promising effect [5, 12, 20, 32]. Rigorous methodological adherence may be experienced as a restriction on practitioners, which could discourage engagement in QI initiatives. However, by strengthening the use of the key features and improving documentation the PDSA projects will be more likely to contribute to IS, including reliable meta-analyses and systematic reviews [10]. This could in return provide QI practitioners with evidence-based knowledge [5, 38]. In this way rigor in performing and documenting QI projects benefits the whole QI community in the long run. It is important that new knowledge becomes readily available and application oriented, in order for practitioners to be motivated to use it. An inherent part of using the PDSA method consists of acknowledging the complexity of creating lasting improvement. Here the scientific ideals about planning, executing, hypothesizing, data managing and documenting with rigor and high quality should serve as inspiration.

Our framework could imply that the presence of all four features will inevitably result in the success of an improvement project. This it clearly not the case. No “magic bullets” exist in QI [39]. QI is about implementing complex projects in complex social contexts. Here adherence to the key methodological recommendations and rigorous documentation can help to ensure better quality and reproducibility. This review can serve as a reminder of these features and how rigor in the individual QI projects can assist the work of IS, which in return can offer new insight for the benefit of practitioners.

## Conclusion

This systematic review documents that substantial methodological challenges remain when reporting from PDSA projects. These challenges pose a problem for the legitimacy of the method. Individual improvement projects should strive to contribute to a scientific foundation for QI by conducting and documenting with a higher rigor. There seems to be a need for methodological improvement when conducting and reporting from QI initiatives.

## Additional files

Additional file 1: Description of variables and coding. (DOCX 24 kb)

Additional file 2: Projects identified in the search that used PDSA method. (DOCX 204 kb)

Additional file 3: Projects identified in search that describes PDSA method in sufficient detail to be included for full analysis for framework. (DOCX 145 kb)

## Data Availability

All data generated or analysed during this review are included in this published article and its supplementary information files.

## Abbreviations

IS: Improvement Science
PDSA: Plan-Do-Study-Act
PRISMA: Preferred Reporting Items for Systematic Reviews and Meta-Analyses
QI: Quality Improvement
SMART: Specific, Measurable, Attainable, Relevant and Timebound
SQUIRE: Standards for QUality Improvement Reporting Excellence
