# Supplementary material for: Can quality improvement improve the quality of care? A systematic review of reported effects and methodological rigor in plan-do-study-act projects
Source: BMC Health Serv Res. 2019 Oct 4;19:683. doi: 10.1186/s12913-019-4482-6 (PMC6778385; doi:10.1186/s12913-019-4482-6)
Supplement: Supplementary file 1 — Description of variables and coding. (DOCX 24 kb) [file 12913_2019_4482_MOESM1_ESM.docx]

**Additional file 1: Description of variables and coding**

| **Variable** | **Description of variable**  (If description is omitted, the variable and coding is seen as self-explanatory) | **Coding of variable** |
| --- | --- | --- |
| **Reach** | Local covers single locations. Regional covers anything above local and up to Nationwide. | 0: Not stated  1: Nationwide  2: Regional  3: Local |
| **Area of healthcare** |  | 0: Not stated  1: Primary care clinic/HMO/GP  2: Department  3: Hospital  4: Community care  5: Public health (or clinic where focus is on public health)  99: Other |
| **Department specialty** |  | 0: Not stated  1: Internal medicine  2: Surgery  3: Paediatrics  4: Oncology  5: Psychiatry  6: Obstetrics or gynaecology  7: Paraclinical  8: Intensive care unit / Emergency department  9: Neurology  10: Otology  99: Other |
| **Supporting framework** | What type of supporting framework did the study claim to use in conjunction with PDSA cycles? | 0: Not stated  1: Lean  2: Six Sigma  3: Institute for Healthcare Improvement (IHI)/Model for Improvement (MFI)  4: Lean-Six Sigma  5: Total Quality Improvement (TQI)  6: Continues Quality Improvement (CQI)  99: Other |
| **Documentation category** | If category 1 or 2 --> Exclude from full analysis against the framework | 1: No details of cycles  2: Themes of cycles but no additional details  3: Details of individual cycles but not stages of cycles  4: Details of cycles including separated information on stages of cycles |
| **Main type of data used** | Type of data used to inform cycles | 1: Quantitative  2: Qualitative  3: Quantitative, supplemented by data on patient/staff satisfaction/experience  4: Both used to inform cycles  5: Quantitative, but data not presented |
| **Scope of QI effort** | Test = creating trust in an intervention through small tests of change  Implementing = an improvement intervention/tool, in which confidence it will lead to improvement already exists, is available. Focus is on integrating it and making it permanent  Spreading = Spreading an implemented improvement intervention  The steps and descriptions must be documented in the PDSA-cycles | 0: Unclear  1: Testing  2: Implementation  3: Spreading  4: Testing and implementing  5: Implementing and spreading  6: Test, implement and spread |
| **Evidence-based improvement** | Is the need for an improvement based in evidence? | 0: No  1: Yes |
| **Origin of knowledge behind change** | Where does the idea that the chosen change intervention will lead to improvement come from? | 0: Not stated  1: External existing knowledge: literature  2: External existing knowledge: guidelines  3: External existing knowledge: benchmarking (best practice, interviews, field visits, observing others etc.)  4: External existing knowledge: previously QI projects  5: Internally developed knowledge: logical thinking (diagrams, cause-effect charts etc.)  6: Internally developed knowledge: creative process  7: Combination of external and internal knowledge |
| **Theoretical rationale** | The presence of an explicit theory formulated, explaining the problems, the assumptions and reasons for why the proposed change is expected to produce the desired outcome | 0: No  1: Yes |
| **Small-scale testing** | Was the change(s) introduced on a scale smaller than an entire department/treatment unit tested, before a full-scale test was begun? | 0: No  1: Yes |
| **Type of scaling when using small scale** | If small tests of change were performed, what was the type of scaling used? | 0: Unclear  1: Increasing  2: Non-increasing |
| **Iterative cycles** | Presence of at least two successive cycles in which lessons from one cycle informed the next. Must be both thematically and functionally linked. | 0: No  1: Yes |
| **Nature of cycles** | What type of cycle was used? | 1: Single isolated cycle  2: Multiple isolated cycles  3: Iterative chain  4: Multiple (isolated) iterative chains  5: Mix of iterative chains and isolated cycles |
| **Several tests of change in a cycle** | Were there several thematically different change processes in a cycle? | 0: No  1: Yes |
| **Data over time** | Continuous measurement over time with regular intervals | 0: No  1: Yes |
| **Type of data over time** | What was the type of visualisation of data over time? | 1: Regular three or more data points  2: Non-regular (before and after or per PDSA cycle)  3: Single data point (after PDSA cycle(s))  4: No quantitative data reported |
| **Type of time series diagram** |  | 0: Not stated  1: Run chart  2: Control chart |
| **Use of Baseline** | Was there a baseline measurement of the primary outcome? | 0: No  1: Yes |
| **Self-reported effect of QI intervention** | Was the QI-project a reported to have resulted in an improvement, and was there a quantitative aim stated pre-intervention?  At least one aim must be achieved to fulfil requirements for category (1), and it must be a sustained improvement. Reaching the point with one data-measurement, followed by a decline from the goal near the end of the intervention does not count as the goal being achieved. | 1: Quantitative aim set and reached  2: No quantitative aim set, improvement registered  3: Quantitative aim set but not reached  4: No quantitative aim and no improvement registered |
