# Supplementary material for: Can quality improvement improve the quality of care? A systematic review of reported effects and methodological rigor in plan-do-study-act projects
Source: BMC Health Serv Res. 2019 Oct 4;19:683. doi: 10.1186/s12913-019-4482-6 (PMC6778385; doi:10.1186/s12913-019-4482-6)
Supplement: Supplementary file 2 — Projects identified in the search that used PDSA method. (DOCX 204 kb) [file 12913_2019_4482_MOESM2_ESM.docx]

**Additional file 2: Studies identified in the search that used PDSA method**

| Author | Country | Reach | Area of Healthcare | Department Specialty | Title | Supporting Framework | Thoroughness of PDSA-description | Was there improvement? |
| --- | --- | --- | --- | --- | --- | --- | --- | --- |
| [1] Afanvi | Togo | Regional | Public Health | N/A | From many deaths to some few cases of drug-resistant tuberculosis: travelling with the systems quality improvement model in Lacs Health District, Togo | Other | 3 - details of individual cycles but not stages of cycles | Positive change - no quantitative aim |
| [2] Ahmad | England | Local | Department | Gyn/obs | Venous thromboembolism capture on electronic systems in obstetrics patients at St Thomas' Hospital | Not stated | 3 - details of individual cycles but not stages of cycles | Positive change - quantitative aim not reached |
| [3] AhmedAwaji | Kingdom of Saudi Arabia | Nationwide | Department | Oncology | Promoting the role of patients in improving hand hygiene compliance amongst health care workers | IHI/MFI | 3 - details of individual cycles but not stages of cycles | Positive change - no quantitative aim |
| [4] Akinbobuyi | England | Local | Department | Intensive care unit / Emergency department | Ensuring timely thromboprophylaxis on a medical assessment unit | Not stated | 3 - details of individual cycles but not stages of cycles | Positive change - quantitative aim not reached |
| [5] Akyuz | England | Regional | Public Health | N/A | Improving the Physical Health Monitoring of City & Hackney Assertive Outreach Service Patients | IHI/MFI | 2 - themes of cycles but no additional details | Quantitative aim was achieved |
| [6] Alhamid | Singapore | Local | Hospital | N/A | Implementing electronic handover: interventions to improve efficiency, safety and sustainability | Not stated | 3 - details of individual cycles but not stages of cycles | Positive change - no quantitative aim |
| [7] Aljaber | Kingdom of Saudi Arabia | Local | PCC/HMO/GP | N/A | Promoting oral health practice among patients with diabetes attending primary health care clinics | Not stated | 4 - details of cycles including seperated information on stages of cycles | Positive change - no quantitative aim |
| [8] AlSaleem | Kingdom of Saudi Arabia | Nationwide | Department | Paraclinic | Reducing the occurrence of errors in a laboratory's specimen receiving and processing department | IHI/MFI | 3 - details of individual cycles but not stages of cycles | Positive change - no quantitative aim |
| [9] Alshieban | Kingdom of Saudi Arabia | Local | Department | Paraclinic | Reducing turnaround time of surgical pathology reports in pathology and laboratory medicine departments | IHI/MFI | 3 - details of individual cycles but not stages of cycles | Quantitative aim was achieved |
| [10] Anderson | Scotland | Local | PCC/HMO/GP | N/A | Quality improvement project using a care bundle approach on the management of the immediate discharge document (IDD) within a single general practice | Not stated | 3 - details of individual cycles but not stages of cycles | Quantitative aim was achieved |
| [11] Andersson | England | Local | Hospital | N/A | Safer fluid prescribing at North Bristol Trust: Bringing practice in line with NICE Guidance with a redesign of the fluid prescription chart | Not stated | 4 - details of cycles including seperated information on stages of cycles | Positive change - no quantitative aim |
| [12] Aragona | USA | Local | Department | Pediatrician | A Quality Improvement Project to Increase Nurse Attendance on Pediatric Family Centered Rounds | IHI/MFI | 3 - details of individual cycles but not stages of cycles | Positive change - quantitative aim not reached |
| [13] Aung | England | Local | Department | Internal Medicine | Less is more: a project to reduce the number of PIMs (potentially inappropriate medications) on an elderly care ward | Not stated | 3 - details of individual cycles but not stages of cycles | Quantitative aim was achieved |
| [14] Bailey | England | Local | Department | Psychiatry | Absconding: Reducing failure to return in adult mental health wards | IHI/MFI | 3 - details of individual cycles but not stages of cycles | Quantitative aim was achieved |
| [15] Bartlett | USA | Local | Hospital | Pediatrician | Improving the efficiency of care for pediatric patients hospitalized with asthma | IHI/MFI | 2 - themes of cycles but no additional details | Quantitative aim was achieved |
| [16] Bays | USA | Local | Department | Internal Medicine | Implementation of disease activity measurement for rheumatoid arthritis patients in an academic rheumatology clinic | Not stated | 3 - details of individual cycles but not stages of cycles | Positive change - no quantitative aim |
| [17] Bell | Scotland | Local | Department | Psychiatry | Succeeding in Sustained Reduction in the use of Restraint using the Improvement Model | IHI/MFI | 3 - details of individual cycles but not stages of cycles | Positive change - no quantitative aim |
| [18] Blackburn | England | Local | Department | Surgery | An enhanced recovery program for elective spinal surgery patients | Not stated | 1 - no details of cycles | Quantitative aim was achieved |
| [19] Bock | Nepal | Local | Hospital | N/A | Improving pneumococcal vaccination rates of medical inpatients in urban Nepal using quality improvement measures | IHI/MFI | 3 - details of individual cycles but not stages of cycles | Positive change - quantitative aim not reached |
| [20] Bray | England | Local | Department | Pediatrician | Improving cranial ultrasound scanning strategy in neonates | Not stated | 3 - details of individual cycles but not stages of cycles | Positive change - quantitative aim not reached |
| [21] Brown | England | Local | Department | Psychiatry | Low stimulus environments: reducing noise levels in continuing care | IHI/MFI | 3 - details of individual cycles but not stages of cycles | Positive change - quantitative aim not reached |
| [22] Brown | England | Regional | Department | Psychiatry | Safer Wards: reducing violence on older people's mental health wards | IHI/MFI | 2 - themes of cycles but no additional details | Quantitative aim was achieved |
| [23] Bryant-Bova | USA | Regional | Department | Paraclinic | Improving chemotherapy ordering process | Not stated | 2 - themes of cycles but no additional details | Positive change - quantitative aim not reached |
| [24] Burchett | USA | Local | Department | Pediatrician | Improving a Urine Culture Callback Follow-up System in a Pediatric Emergency Department | IHI/MFI | 3 - details of individual cycles but not stages of cycles | Quantitative aim was achieved |
| [25] Calderwood | USA | Local | Department | Surgery | A Plan-Do-Study-Act Approach to Improving Bowel Preparation Quality | Not stated | 2 - themes of cycles but no additional details | Positive change - quantitative aim not reached |
| [26] Chartier | Canada | Local | Department | Intensive care unit / Emergency department | Improving Emergency Department flow through optimized bed utilization | Other | 3 - details of individual cycles but not stages of cycles | Positive change - quantitative aim not reached |
| [27] Cohen | USA | Local | Hospital | N/A | Influenza vaccination rates for hospitalised patients: a multiyear quality improvement effort | IHI/MFI | 3 - details of individual cycles but not stages of cycles | Positive change - quantitative aim not reached |
| [28] Cohen | USA | Local | Department | Intensive care unit / Emergency department | A quality improvement project to decrease emergency department and medical intensive care unit transfer times | Not stated | 1 - no details of cycles | Quantitative aim was achieved |
| [29] Cooper | England | Local | Department | Psychiatry | Improving the rate of Patient Feedback for a Later Life Mental Health Liaison Team | Not stated | 3 - details of individual cycles but not stages of cycles | Quantitative aim was achieved |
| [30] Cottney | England | Regional | Department | Psychiatry | Using league tables to reduce missed dose medication errors on mental healthcare of older people wards | Not stated | 3 - details of individual cycles but not stages of cycles | Positive change - no quantitative aim |
| [31] Crosby | USA | Local | Department | Internal Medicine | Implementation of a Process for Initial Transcranial Doppler Ultrasonography in Children With Sickle Cell Anemia | Not stated | 1 - no details of cycles | Quantitative aim was achieved |
| [32] Croxford | England | Local | Department | Psychiatry | Introduction of a Venous Thromboembolism Prophylaxis Protocol for Older Adult Psychiatric Patients | Not stated | 4 - details of cycles including seperated information on stages of cycles | Positive change - no quantitative aim |
| [33] Curatolo | France | Local | Department | Surgery | Reducing medication errors at admission: 3 cycles to implement, improve and sustain medication reconciliation | Not stated | 3 - details of individual cycles but not stages of cycles | Positive change - no quantitative aim |
| [34] Dandoy | USA | Local | Department | Pediatrician | Sustained reductions in time to antibiotic delivery in febrile immunocompromised children: results of a quality improvement collaborative | IHI/MFI | 1 - no details of cycles | Quantitative aim was achieved |
| [35] DeCristofano | Argentina | Local | Department | Pediatrician | Implementation of a Ventilator-Associated Pneumonia Prevention Bundle in a Single PICU | Not stated | 1 - no details of cycles | Quantitative aim was achieved |
| [36] Dewey | USA | Not stated | Not stated | Not stated | Developing a Deep Brain Stimulation Neuromodulation Network for Parkinson Disease, Essential Tremor, and Dystonia: Report of a Quality Improvement Project | Not stated | 1 - no details of cycles | Positive change - no quantitative aim |
| [37] Donaldson | England | Local | Department | Intensive care unit / Emergency department | Improving quality in a national intestinal failure unit: Greater efficiency, improved access and reduced mortality | IHI/MFI | 1 - no details of cycles | Quantitative aim was achieved |
| [38] Donnelly | Northern Ireland | Local | Department | Pediatrician | Improving reporting of critical incidents through education and involvement | Not stated | 3 - details of individual cycles but not stages of cycles | Positive change - no quantitative aim |
| [39] Dorrington | Australia | Local | Public Health | N/A | Increasing Pap smear rates at an urban Aboriginal Community Controlled Health Service through translational research and continuous quality improvement | CQI | 2 - themes of cycles but no additional details | Positive change - no quantitative aim |
| [40] Dummett | USA | Regional | Hospital | N/A | Incorporating an Early Detection System Into Routine Clinical Practice in Two Community Hospitals | IHI/MFI | 1 - no details of cycles | Positive change - no quantitative aim |
| [41] Dunbar | England | Local | PCC/HMO/GP | Internal Medicine | Mortality meetings in geriatric medicine: strategies for improvement | Not stated | 3 - details of individual cycles but not stages of cycles | Positive change - no quantitative aim |
| [42] Dykes | USA | Local | Department | Pediatrician | Improving pediatric Inflammatory Bowel Disease (IBD) follow-up | IHI/MFI | 3 - details of individual cycles but not stages of cycles | Quantitative aim was achieved |
| [43] Fontanez-Nieves | USA | Local | Department | Pediatrician | Prevention of unplanned extubations in neonates through process standardization | Not stated | 3 - details of individual cycles but not stages of cycles | Quantitative aim was achieved |
| [44] Frost | England | Local | Hospital | N/A | Reducing the overuse of betahCG measurements in the emergency gynaecology clinic | Not stated | 4 - details of cycles including seperated information on stages of cycles | Positive change - no quantitative aim |
| [45] Goodman | USA | Local | Department | Oncology | Reduction of Inappropriate Prophylactic Pegylated Granulocyte Colony-Stimulating Factor Use for Patients With Non-Small-Cell Lung Cancer Who Receive Chemotherapy: An ASCO Quality Training Program Project of the Cleveland Clinic Taussig Cancer Institute | IHI/MFI | 2 - themes of cycles but no additional details | Positive change - no quantitative aim |
| [46] Goulding | England | Local | Hospital | Intensive care unit / Emergency department | Improving critical care discharge summaries: a collaborative quality improvement project using PDSA | IHI/MFI | 3 - details of individual cycles but not stages of cycles | Failure, change in unwanted direction |
| [47] Guo | Canada | Local | Hospital | N/A | Let's Talk About Sex! - Improving sexual health for patients in stroke rehabilitation | Not stated | 3 - details of individual cycles but not stages of cycles | Positive change - no quantitative aim |
| [48] Hale | Scotland | Local | Department | Internal Medicine | Developing a ward round checklist to improve patient safety | Not stated | 3 - details of individual cycles but not stages of cycles | Positive change - no quantitative aim |
| [49] Hall | England | Local | Community Care | N/A | Improving the safety of prescriptions of domperidone in primary care: implementing MHRA advice | Not stated | 3 - details of individual cycles but not stages of cycles | Quantitative aim was achieved |
| [50] Hanison | England | Local | Department | Intensive care unit / Emergency department | A multifaceted approach to prevention of delirium on intensive care | Not stated | 3 - details of individual cycles but not stages of cycles | Positive change - no quantitative aim |
| [51] Hatoun | USA | Local | Department | Pediatrician | Increasing Medication Possession at Discharge for Patients With Asthma: The Meds-in-Hand Project | IHI/MFI | 3 - details of individual cycles but not stages of cycles | Quantitative aim was achieved |
| [52] Haydar | USA | Local | Department | Intensive care unit / Emergency department | Sustainable Mechanism to Reduce Emergency Department (ED) Length of Stay: The Use of ED Holding (ED Transition) Orders to Reduce ED Length of Stay | Not stated | 1 - no details of cycles | Positive change - no quantitative aim |
| [53] Hayes | USA | Local | Hospital | Pediatrician | A Quality Improvement Project to Improve Family Recognition of Medical Team Member Roles | Not stated | 3 - details of individual cycles but not stages of cycles | Positive change - quantitative aim not reached |
| [54] Hendricks | USA | Local | Department | Internal Medicine | Assessing referrals and improving information availability for consultations in an academic endocrinology clinic | Other | 3 - details of individual cycles but not stages of cycles | Positive change - quantitative aim not reached |
| [55] Hendricks | USA | Local | Department | Oncology | Improving adherence with oral antiemetic agents in patients with breast cancer receiving chemotherapy | Not stated | 1 - no details of cycles | Positive change - quantitative aim not reached |
| [56] Hermon | Wales | Local | Department | Intensive care unit / Emergency department | Improving compliance with central venous catheter care bundles using electronic records | CQI | 1 - no details of cycles | Positive change - no quantitative aim |
| [57] Hill | USA | Local | Department | Psychiatry | Measurable results: Reducing staff injuries on a specialty psychiatric unit for patients with developmental disabilities | IHI/MFI | 1 - no details of cycles | Positive change - no quantitative aim |
| [58] Holmes | USA | Local | Department | Pediatrician | Rooming-In to Treat Neonatal Abstinence Syndrome: Improved Family-Centered Care at Lower Cost | Not stated | 3 - details of individual cycles but not stages of cycles | Positive change - no quantitative aim |
| [59] Izudi | Uganda | Local | Hospital | N/A | Quality improvement interventions for early HIV infant diagnosis in northeastern uganda | Other | 2 - themes of cycles but no additional details | Quantitative aim was achieved |
| [60] Jobson | USA | Local | Department | Intensive care unit / Emergency department | Decreasing time to antibiotics in febrile patients with central lines in the emergency department | Not stated | 1 - no details of cycles | Quantitative aim was achieved |
| [61] Johnson | USA | Local | Department | Pediatrician | Toward High-Value Care: A Quality Improvement Initiative to Reduce Unnecessary Repeat Complete Blood Counts and Basic Metabolic Panels on a Pediatric Hospitalist Service | Not stated | 2 - themes of cycles but no additional details | Quantitative aim was achieved |
| [62] Jones | USA | Local | Department | Surgery | Beyond best practice: Implementing a unit-based CLABSI project | IHI/MFI | 3 - details of individual cycles but not stages of cycles | Positive change - no quantitative aim |
| [63] Kaur | Canada | Local | Department | Internal Medicine | Adressing Opiod-Associated constipation using quality oncology practice initiative scores and plan-do-study-act cycles | Not stated | 2 - themes of cycles but no additional details | Positive change - no quantitative aim |
| [64] Kulik | England | Local | Department | Other | Role of peer support workers in improving patient experience in Tower Hamlets Specialist Addiction Unit | IHI/MFI | 4 - details of cycles including seperated information on stages of cycles | Quantitative aim was achieved |
| [65] Kumar | India | Local | Hospital | Other | Improving medical stores management through automation and effective communication | Not stated | 2 - themes of cycles but no additional details | Positive change - no quantitative aim |
| [66] Li | USA | Local | Department | Surgery | Implementation and Results of a Percutaneous Renal Allograft Biopsy Protocol to Reduce Complication Rate | Not stated | 4 - details of cycles including seperated information on stages of cycles | Positive change - no quantitative aim |
| [67] Looper | USA | Local | Department | Oncology | Best Practices for Chemotherapy Administration in Pediatric Oncology: Quality and Safety Process Improvements (2015) | IHI/MFI | 3 - details of individual cycles but not stages of cycles | Positive change - no quantitative aim |
| [68] Low | Singapore | Local | Hospital | Psychiatry | Reducing transfers of psychiatric inpatients to emergency rooms of general hospitals in Singapore: a clinical practice improvement project | IHI/MFI | 1 - no details of cycles | Quantitative aim was achieved |
| [69] Maqbool | Canada | Local | Hospital | Surgery | Importance of patient-centred signage and navigation guide in an orthopaedic and plastics clinic | Not stated | 3 - details of individual cycles but not stages of cycles | Positive change - no quantitative aim |
| [70] Margalit | USA | Local | Department | Oncology | Ensuring head and neck oncology patients receive recommended pretreatment dental evaluations | IHI/MFI | 1 - no details of cycles | Positive change - no quantitative aim |
| [71] May-Miller | England | Local | Hospital | N/A | Improving the quality of discharge summaries: implementing updated Academy of Medical Royal Colleges standards at a district general hospital | Not stated | 3 - details of individual cycles but not stages of cycles | Positive change - no quantitative aim |
| [72] McCormack | England | Local | Hospital | Psychiatry | Improving the uptake and comprehensiveness of bedside cognitive testing amongst liaison psychiatrists over an eight-month period | Not stated | 3 - details of individual cycles but not stages of cycles | Positive change - no quantitative aim |
| [73] Mgonja | USA | Local | PCC/HMO/GP | N/A | Postpartum Depression Screening at Well-Child Appointments: A Quality Improvement Project | IHI/MFI | 3 - details of individual cycles but not stages of cycles | Positive change - no quantitative aim |
| [74] Minhas | Singapore | Local | Hospital | N/A | Redesigning case selection methods to improve clinical review of inpatient medical records | Other | 3 - details of individual cycles but not stages of cycles | Positive change - no quantitative aim |
| [75] Montella | USA | Local | Department | Gyn/obs | Improving the Rate of Colposcopy in an Urban Population of Patients With Known Abnormal Pap Smears | IHI/MFI | 1 - no details of cycles | Positive change - quantitative aim not reached |
| [76] Mookadam | USA | Local | PCC/HMO/GP | N/A | Simple Interventions Improve the Quality of a Missed Lab Appointment Process | Not stated | 3 - details of individual cycles but not stages of cycles | Positive change - no quantitative aim |
| [77] Murphy | Republic of Ireland | Local | Community Care | N/A | A Quality Improvement Initiative in Community Mental Health in the Republic of Ireland | IHI/MFI | 3 - details of individual cycles but not stages of cycles | No change |
| [78] Mustafa | Qatar | Local | Department | Pediatrician | Understanding and overcoming barriers to timely discharge from the pediatric units | Not stated | 4 - details of cycles including seperated information on stages of cycles | Positive change - no quantitative aim |
| [79] Nelson | England | Local | Hospital | Intensive care unit / Emergency department | Inpatient Falls: Improving assessment, documentation, and management | Not stated | 3 - details of individual cycles but not stages of cycles | Positive change - no quantitative aim |
| [80] Nuti | Wales | Local | Department | Pediatrician | Improving compliance with iron infusion therapy in the treatment of chronic anemia in haemodialysis patients with chronic kidney disease | Not stated | 4 - details of cycles including seperated information on stages of cycles | Positive change - no quantitative aim |
| [81] Ozekcin | USA | Local | Hospital | N/A | Simulation education: early identification of patient physiologic deterioration by acute care nurses | IHI/MFI | 4 - details of cycles including seperated information on stages of cycles | Positive change - no quantitative aim |
| [82] Partridge | England | Local | Department | Surgery | Improving patient reported outcome measures (PROMs) in total knee replacement by changing implant and preserving the infrapatella fatpad: a quality improvement project | Not stated | 3 - details of individual cycles but not stages of cycles | Positive change - no quantitative aim |
| [83] Peh | Singapore | Local | Hospital | Intensive care unit / Emergency department | Eliminating guidewire retention during ultrasound guided central venous catheter insertion via an educational program, a modified CVC set, and a drape with reminder stickers | Not stated | 3 - details of individual cycles but not stages of cycles | Quantitative aim was achieved |
| [84] Prager | USA | Local | Hospital | Pediatrician | Improving operative flow during pediatric airway evaluation: a quality-improvement initiative | Not stated | 2 - themes of cycles but no additional details | Positive change - quantitative aim not reached |
| [85] Reilly | England | Local | Department | Psychiatry | Improving ward environments and developing skills for discharge with the implementation of self-catering on a low secure forensic unit | Not stated | 3 - details of individual cycles but not stages of cycles | Positive change - quantitative aim not reached |
| [86] Reynolds | UK | Regional | Hospital | N/A | Improving feedback on junior doctors' prescribing errors: mixed-methods evaluation of a quality improvement project | Not stated | 2 - themes of cycles but no additional details | Positive change - no quantitative aim |
| [87] Riblet | USA | Local | Department | Oncology | A clinical care pathway to improve the acute care of patients with glioma | Lean-Six Sigma | 2 - themes of cycles but no additional details | Positive change - no quantitative aim |
| [88] Roberts | England | Local | Department | Surgery | Improving theatre efficiency and utilisation through early identification of trauma patients and enhanced communication between teams | Not stated | 4 - details of cycles including seperated information on stages of cycles | Positive change - no quantitative aim |
| [89] Rostami | England | Nationwide | Other | Not stated | Learning from the design development and implementation of the medication safety thermometer | Not stated | 4 - details of cycles including seperated information on stages of cycles | Positive change - no quantitative aim |
| [90] Salman | Wales | Local | Hospital | Internal Medicine | Alcohol detoxification in Ysbyty Gwynedd: Two small sips or one big gulp? Two-step screening more reliable for identification of alcohol dependency syndrome at risk of delirium tremens for routine care | Not stated | 3 - details of individual cycles but not stages of cycles | Positive change - no quantitative aim |
| [91] Salstrom | USA | Local | Hospital | Pediatrician | Pediatric patients who receive antibiotics for fever and neutropenia in less than 60 min have decreased intensive care needs | Lean | 3 - details of individual cycles but not stages of cycles | Positive change - quantitative aim not reached |
| [92] Samaan | USA | Regional | PCC/HMO/GP | Pediatrician | Implementation of a preventive services bundle in academic pediatric primary care centers | IHI/MFI | 2 - themes of cycles but no additional details | Positive change - quantitative aim not reached |
| [93] Samji | Canada | Local | Department | Surgery | Quality improvement initiative to increase consistent use of intraluminal contrast in the identification of anastomotic bowel leaks on computed tomography using plan-do-study-act service approach | Not stated | 3 - details of individual cycles but not stages of cycles | Positive change - no quantitative aim |
| [94] Savarino | USA | Local | Hospital | Pediatrician | Improving Clinical Remission Rates in Pediatric Inflammatory Bowel Disease with Previsit Planning | IHI/MFI | 3 - details of individual cycles but not stages of cycles | Quantitative aim was achieved |
| [95] Schorr | USA | Regional | Hospital | N/A | Implementation of a multicenter performance improvement program for early detection and treatment of severe sepsis in general medical-surgical wards | IHI/MFI | 1 - no details of cycles | Positive change - no quantitative aim |
| [96] Shahnazarian | USA | Local | Hospital | N/A | Hepatitis C: improving the quality of screening in a community hospital by implementing an electronic medical record intervention | Not stated | 2 - themes of cycles but no additional details | Positive change - no quantitative aim |
| [97] Shaikh | USA | Local | Hospital | Pediatrician | Triple Duty: Integrating Graduate Medical Education With Maintenance of Board Certification to Improve Clinician Communication at Hospital Discharge | Not stated | 2 - themes of cycles but no additional details | Positive change - no quantitative aim |
| [98] Singh | England | Local | Department | Internal Medicine | A quality improvement initiative on the management of osteoporosis in older people with Parkinsonism | IHI/MFI | 3 - details of individual cycles but not stages of cycles | Positive change - quantitative aim not reached |
| [99] Singh | Wales | Local | Hospital | Not stated | Reducing inpatient falls in a 100% single room elderly care environment: evaluation of the impact of a systematic nurse training programme on falls risk assessment (FRA) | Not stated | 2 - themes of cycles but no additional details | Quantitative aim was achieved |
| [100] Stark | England | Regional | Department | Surgery | Improving patient flow in pre-operative assessment | Lean | 1 - no details of cycles | Positive change - no quantitative aim |
| [101] Subramanyam | USA | Local | Department | Pediatrician | Infusion Medication error reduction by tow-person verification: A quality improvement initiative | IHI/MFI | 2 - themes of cycles but no additional details | Quantitative aim was achieved |
| [102] Sudhanthar | USA | Local | Department | Pediatrician | Improving viable low cost generic medication prescription rate in primary care pediatric practice | Not stated | 3 - details of individual cycles but not stages of cycles | Positive change - quantitative aim not reached |
| [103] Sudhanthar | USA | Local | Department | Pediatrician | Improving validated depression screen among adolescent population in primary care practice using electronic health records (EHR) | Not stated | 3 - details of individual cycles but not stages of cycles | Quantitative aim was achieved |
| [104] Tan | Singapore | Local | Department | Oncology | Using Quality Improvement Methods and Time-Driven Activity-Based Costing to Improve Value-Based Cancer Care Delivery at a Cancer Genetics Clinic | Not stated | 3 - details of individual cycles but not stages of cycles | Quantitative aim was achieved |
| [105] Thomas | USA | Local | Department | Intensive care unit / Emergency department | Impact of a Quality Improvement Intervention to Increase Brief Alcohol and Drug Interventions on a Level I Trauma Service | Not stated | 2 - themes of cycles but no additional details | Positive change - no quantitative aim |
| [106] Thomassen | Zanzibar | Local | Department | Intensive care unit / Emergency department | Emergency medicine in Zanzibar: the effect of system changes in the emergency department | Not stated | 3 - details of individual cycles but not stages of cycles | Positive change - no quantitative aim |
| [107] Titsworth | USA | Local | Department | Neurology | A prospective time-series quality improvement trial of a standardized analgesia protocol to reduce postoperative pain among neurosurgery patients | Not stated | 2 - themes of cycles but no additional details | Positive change - no quantitative aim |
| [108] Tripathi | USA | Local | Department | Pediatrician | Implementation of patient-centered bedside rounds in the pediatric intensive care unit | Not stated | 4 - details of cycles including seperated information on stages of cycles | Positive change - no quantitative aim |
| [109] Ugarte | England | Local | Department | Other | Waiting time reduction in intravitreal clinics by optimization of appointment scheduling: balancing demand and supply | IHI/MFI | 1 - no details of cycles | Positive change - no quantitative aim |
| [110] Vetter | USA | Local | PCC/HMO/GP | N/A | The Influence of Clinical Decision Support on Diagnostic Accuracy in Nurse Practitioners | Not stated | 1 - no details of cycles | Positive change - no quantitative aim |
| [111] Wale | Canada | Regional | Public Health | N/A | New approaches to infection prevention and control: implementing a risk-based model regionally | Not stated | 1 - no details of cycles | Positive change - no quantitative aim |
| [112] Walton | England | Regional | Department | Internal Medicine | Improving the quality of handover by addressing handover culture and introducing a new, multi-disciplinary, team-based handover meeting | Not stated | 3 - details of individual cycles but not stages of cycles | Positive change - no quantitative aim |
| [113] Ward | England | Local | Hospital | Surgery | Standardising the organisation of clinical equipment on surgical wards at North Bristol NHS Trust: a quality improvement initiative | Not stated | 3 - details of individual cycles but not stages of cycles | Positive change - no quantitative aim |
| [114] Wiemann | USA | Local | Hospital | Pediatrician | Integrating an EMR-based Transition Planning Tool for CYSHCN at a Children's Hospital: A Quality Improvement Project to Increase Provider Use and Satisfaction | IHI/MFI | 3 - details of individual cycles but not stages of cycles | Positive change - no quantitative aim |
| [115] Wittkugel | USA | Local | Hospital | Surgery | Development of a nurse-assisted preanesthesia evaluation program for pediatric outpatient anesthesia | IHI/MFI | 1 - no details of cycles | Quantitative aim was achieved |
| [116] Wrenn | USA | Local | PCC/HMO/GP | N/A | Using a self-service kiosk to identify behavioural health needs in a primary care clinic serving an urban, underserved population | Not stated | 2 - themes of cycles but no additional details | Positive change - no quantitative aim |
| [117] Wu | USA | Regional | Hospital | Pediatrician | A Quality Improvement Collaborative to Improve the Discharge Process for Hospitalized Children | IHI/MFI | 1 - no details of cycles | Positive change - quantitative aim not reached |
| [118] Yager | Northern Ireland | Local | Hospital | Pediatrician | Quality improvement utilizing in-situ simulation for a dual-hospital pediatric code response team | IHI/MFI | 2 - themes of cycles but no additional details | Positive change - no quantitative aim |
| [119] Yelland | Australia | Local | Hospital | Gyn/obs | Bridging the language gap: a co-designed quality improvement project to engage professional interpreters for women duing labour | Not stated | 2 - themes of cycles but no additional details | Positive change - no quantitative aim |
| [120] Zuccarelli | USA | Local | Hospital | Pediatrician | Reducing after-hours prescription refill requests | Not stated | 2 - themes of cycles but no additional details | Positive change - no quantitative aim |

1 Afanvi KA. From many deaths to some few cases of drug-resistant tuberculosis: travelling with the systems quality improvement model in Lacs Health District, Togo. *BMJ Qual Improv Reports* 2015;**4**:u201413.w1473.

2 Ahmad AN, Leyla Byrne M, Imambaccus N, *et al.* Venous thromboembolism capture on electronic systems in obstetrics patients at St Thomas’ Hospital. *BMJ Qual Improv Reports* 2016;**5**:u212405.w5122.

3 Ahmed Awaji M, Al-Surimi K. Promoting the role of patients in improving hand hygiene compliance amongst health care workers. *BMJ Qual Improv Reports* 2016;**5**:u210787.w4336.

4 Akinbobuyi O, shalders louise, Nokes T. Ensuring timely thromboprophylaxis on a Medical Assessment Unit. *BMJ Qual Improv Reports* 2016;**5**:u212414.w4934.

5 Akyuz E, jain amit, phelan declan, *et al.* Improving the Physical Health Monitoring of City &amp; Hackney Assertive Outreach Service Patients. *BMJ Qual Improv Reports* 2016;**5**:u210108.w4174.

6 Alhamid SM, Lee DX-Y, Wong HM, *et al.* Implementing electronic handover: interventions to improve efficiency, safety and sustainability. *Int J Qual Heal care J Int Soc Qual Heal Care* 2016;**28**:608–14.

7 Aljaber A, Al-Surimi K. Promoting oral health practice among patients with diabetes attending primary health care clinics. *BMJ Qual Improv reports* 2015;**4**:10.1136/bmjquality.u209172.w3737. eCollection 2015.

8 Al Saleem N, Al-Surimi K. Reducing the occurrence of errors in a laboratory’s specimen receiving and processing department. *BMJ Qual Improv reports* 2016;**5**:u211474.w4624. eCollection 2016.

9 Alshieban S, Al-Surimi K. Reducing turnaround time of surgical pathology reports in pathology and laboratory medicine departments. *BMJ Qual Improv Reports* 2015;**4**:u209223.w3773.

10 Anderson C, McNab D. Quality improvement project using a care bundle approach on the management of the immediate discharge document (IDD) within a single general practice. *BMJ Qual Improv reports* 2015;**4**:10.1136/bmjquality.u204819.w3337. eCollection 2015.

11 Andersson J, Bull T, Paul D, *et al.* Safer fluid prescribing at North Bristol Trust: Bringing practice in line with NICE Guidance with a redesign of the fluid prescription chart. *BMJ Qual Improv Reports* 2015;**4**:u203816.w1911.

12 Aragona E, Ponce-Rios J, Garg P, *et al.* A Quality Improvement Project to Increase Nurse Attendance on Pediatric Family Centered Rounds. *J Pediatr Nurs* 2016;**31**:e3–9.

13 Aung TH, Judith Beck A, Siese T, *et al.* Less is more: a project to reduce the number of PIMs (potentially inappropriate medications) on an elderly care ward. *BMJ Qual Improv reports* 2016;**5**:1–4.

14 Bailey J, Page B, Ndimande N, *et al.* Absconding: reducing failure to return in adult mental health wards. *BMJ Qual Improv Reports* 2016;**5**:u209837.w5117.

15 Bartlett KW, Parente VM, Morales V, *et al.* Improving the Efficiency of Care for Pediatric Patients Hospitalized With Asthma. *Hosp Pediatr* 2017;**7**:31–8.

16 Bays A, Wahl E, Daikh DI, *et al.* Implementation of disease activity measurement for rheumatoid arthritis patients in an academic rheumatology clinic. *BMC Health Serv Res* 2016;**16**:384.

17 Bell A, Gallacher N. Succeeding in Sustained Reduction in the use of Restraint using the Improvement Model. *BMJ Qual Improv Reports* 2016;**5**:u211050.w4430.

18 Blackburn J, Madhavan P, Leung YL, *et al.* An enhanced recovery program for elective spinal surgery patients. *J Clin Outcomes Manag* 2016;**23**:462–9.

19 Bock A, Chintamaneni K, Rein L, *et al.* Improving pneumococcal vaccination rates of medical inpatients in urban Nepal using quality improvement measures. *BMJ Qual Improv Reports* 2016;**5**:u212047.w4835.

20 Bray L. Improving cranial ultrasound scanning strategy in neonates. *BMJ Qual Improv Reports* 2016;**5**:u210346.w4219.

21 Brown J, Fawzi W, Shah A, *et al.* Low stimulus environments: reducing noise levels in continuing care. *BMJ Qual Improv Reports* 2016;**5**:u207447.w4214.

22 Brown J, Fawzi W, McCarthy C, *et al.* Safer Wards: reducing violence on older people’s mental health wards. *BMJ Qual Improv reports* 2015;**4**:1–5.

23 Bryant-Bova JN. Improving Chemotherapy Ordering Process. *J Oncol Pract* 2016;**12**:e248–56.

24 Burchett P, Harpin S, Petersen-Smith A, *et al.* Improving a Urine Culture Callback Follow-up System in a Pediatric Emergency Department. *J Pediatr Heal Care* 2015;**29**:518–25.

25 Calderwood AH, Mahoney EM, Jacobson BC. A Plan-Do-Study-Act Approach to Improving Bowel Preparation Quality. *Am J Med Qual* 2017;**32**:194–200.

26 Chartier L, Josephson T, Bates K, *et al.* Improving emergency department flow through Rapid Medical Evaluation unit. *BMJ Qual Improv Reports* 2015;**4**:u206156.w2663.

27 Cohen ES, Ogrinc G, Taylor T, *et al.* Influenza vaccination rates for hospitalised patients: A multiyear quality improvement effort. *BMJ Qual Saf* 2015;**24**:221–7.

28 Cohen R, Kennedy H, Amitrano B, *et al.* A quality improvement project to decrease emergency department and medical intensive care unit transfer times. *J Crit* 2015;**30**:1331–7.

29 Cooper D. Improving the rate of Patient Feedback for a Later Life Mental Health Liaison Team. *BMJ Qual Improv Reports* 2016;**5**:u210384.w4457.

30 Cottney A. Using league tables to reduce missed dose medication errors on mental healthcare of older people wards. *BMJ Qual Improv Reports* 2015;**4**:u204237.w3567.

31 Crosby LE, Joffe NE, Davis B, *et al.* Implementation of a Process for Initial Transcranial Doppler Ultrasonography in Children With Sickle Cell Anemia. *Am J Prev Med* 2016;**51**:S10-6.

32 Croxford A, Clare A, McCurdy K. Introduction of a Venous Thromboembolism Prophylaxis Protocol for Older Adult Psychiatric Patients. *BMJ Qual Improv Reports* 2015;**4**:u205852.w3226.

33 Curatolo N, Gutermann L, Devaquet N, *et al.* Reducing medication errors at admission: 3 cycles to implement, improve and sustain medication reconciliation. *Int J Clin Pharm* 2014;**37**:113–20.

34 Dandoy CE, Hariharan S, Weiss B, *et al.* Sustained reductions in time to antibiotic delivery in febrile immunocompromised children: Results of a quality improvement collaborative. *BMJ Qual Saf* 2016;**25**:100–9.

35 De Cristofano A, Peuchot V, Canepari A, *et al.* Implementation of a ventilator-associated pneumonia prevention bundle in a single PICU. *Pediatr Crit Care Med* 2016;**17**:451–6.

36 Dewey RB, O’Suilleabhain PE, Sanghera M, *et al.* Developing a deep brain stimulation neuromodulationnetwork for Parkinson disease, essential tremor, and dystonia: Report of a quality improvement project. *PLoS One* 2016;**11**:1–11.

37 Donaldson E, Taylor M, Abraham A, *et al.* OC-039 Improving Quality In A National Intestinal Failure Unit: Greater Efficiency, Improved Access, Reduced Mortality. *Gut* 2014;**63**:A19.1-A19.

38 Donnelly P. Improving reporting of critical incidents through education and involvement. *BMJ Qual Improv Reports* 2015;**4**:u206996.w3776.

39 Dorrington MS, Herceg A, Douglas K, *et al.* Increasing Pap smear rates at an urban Aboriginal Community Controlled Health Service through translational research and continuous quality improvement. *Aust J Prim Health* 2015;**21**:417–22.

40 Dummett BA, Adams C, Scruth E, *et al.* Incorporating an Early Detection System Into Routine Clinical Practice in Two Community Hospitals. *J Hosp Med* 2016;**11**:S25–31.

41 Dunbar J, George J. Mortality meetings in geriatric medicine: strategies for improvement. *BMJ Qual Improv Reports* 2015;**4**:u202625.w3247.

42 Dykes D, Williams E, Margolis P, *et al.* Improving pediatric Inflammatory Bowel Disease (IBD) follow-up. *BMJ Qual Improv Reports* 2016;**5**:u208961.w3675.

43 Fontánez-Nieves TD, Frost M, Anday E, *et al.* Prevention of unplanned extubations in neonates through process standardization. *J Perinatol* 2016;**36**:469–73.

44 Frost L. Reducing the overuse of βhCG measurements in the emergency gynaecology clinic. *BMJ Qual Improv Reports* 2016;**5**:u210039.w4218.

45 Martin Goodman L, Moeller MB, Azzouqa A-G, *et al.* Reduction of Inappropriate Prophylactic Pegylated Granulocyte Colony-Stimulating Factor Use for Patients With Non–Small-Cell Lung Cancer Who Receive Chemotherapy: An ASCO Quality Training Program Project of the Cleveland Clinic Taussig Cancer Institute. *J Oncol Pract* 2016;**12**:e101–7.

46 Goulding L, Parke H, Maharaj R, *et al.* Improving critical care discharge summaries: a collaborative quality improvement project using PDSA. *BMJ Qual Improv Reports* 2015;**4**:u203938.w3268.

47 Guo M, Bosnyak S, Bontempo T, *et al.* Let’s Talk About Sex! - Improving sexual health for patients in stroke rehabilitation. *BMJ Qual Improv Reports* 2015;**4**:u207288.w2926.

48 Hale G, McNab D. Developing a ward round checklist to improve patient safety. *BMJ Qual Improv Reports* 2015;**4**:u204775.w2440-u204775.w2440.

49 Hall W. Improving the safety of prescriptions of domperidone in primary care: implementing MHRA advice. *BMJ Qual Improv Reports* 2016;**5**:u209711.w4039.

50 Hanison J, Conway D. A multifaceted approach to prevention of delirium on intensive care. *BMJ Qual Improv Reports* 2015;**4**:u209656.w4000.

51 Hatoun J, Bair-Merritt M, Cabral H, *et al.* Increasing Medication Possession at Discharge for Patients With Asthma: The Meds-in-Hand Project. *Pediatrics* 2016;**137**:e20150461–e20150461.

52 Haydar SA, Strout TD, Baumann MR. Sustainable Mechanism to Reduce Emergency Department (ED) Length of Stay: The Use of ED Holding (ED Transition) Orders to Reduce ED Length of Stay. *Acad Emerg Med* 2016;**23**:776–85.

53 Hayes RM, Wickline A, Hensley C, *et al.* A Quality Improvement Project to Improve Family Recognition of Medical Team Member Roles. *Hosp Pediatr* 2015;**5**:480–6.

54 Hendrickson CD, Saini S, Pothuloori A, *et al.* Assessing Referrals and Improving Information Availability for Consultations in an Academic Endocrinology Clinic. Endocr. Pract. 2017;**23**:190–8.

55 Hendricks CB. Improving adherence with oral antiemetic agents in patients with breast cancer receiving chemotherapy. *J Oncol Pr* 2015;**11**:216–8.

56 Hermon A, Pain T, Beckett P, *et al.* Improving compliance with central venous catheter care bundles using electronic records. *Nurs Crit Care* 2015;**20**:196–203.

57 Hill AK, Lind MA, Tucker D, *et al.* Measurable results: Reducing staff injuries on a specialty psychiatric unit for patients with developmental disabilities. *Work* 2015;**51**:99–111.

58 Holmes A V., Atwood EC, Whalen B, *et al.* Rooming-In to Treat Neonatal Abstinence Syndrome: Improved Family-Centered Care at Lower Cost. *Pediatrics* 2016;**137**:e20152929–e20152929.

59 Izudi J, Akot A, Kisitu GP, *et al.* Quality Improvement Interventions for Early HIV Infant Diagnosis in Northeastern Uganda. *Biomed Res Int* 2016;**2016**.

60 Jobson M, Sandrof M, Valeriote T, *et al.* Decreasing Time to Antibiotics in Febrile Patients With Central Lines in the Emergency Department. *Pediatrics* 2015;**135**:e187–95.

61 Johnson DP, Lind C, Parker SES, *et al.* Toward High-Value Care: A Quality Improvement Initiative to Reduce Unnecessary Repeat Complete Blood Counts and Basic Metabolic Panels on a Pediatric Hospitalist Service. *Hosp Pediatr* 2016;**6**:1–8.

62 Jones CM, Stewart C, Roszell SS. Beyond best practice implementing a unit-based CLABSI project. *J Nurs Care Qual* 2015;**30**:24–30.

63 Kaur V, Haider S, Sasapu A, *et al.* Addressing Opioid-Associated Constipation Using Quality Oncology Practice Initiative Scores and Plan-Do-Study-Act Cycles. *J Oncol Pract* 2017;**13**:e91–7.

64 Kulik W, Shah A. Role of peer support workers in improving patient experience in Tower Hamlets Specialist Addiction Unit. *BMJ Qual Improv Reports* 2016;**5**:u205967.w2458.

65 Kumar A, Cariappa MP, Marwaha V, *et al.* Improving medical stores management through automation and effective communication. *Med J Armed Forces India* 2016;**72**:61–6.

66 Li CH, Traube LE, Lu DS, *et al.* Implementation and Results of a Percutaneous Renal Allograft Biopsy Protocol to Reduce Complication Rate. *J Am …* 2016;**13**:549–53.

67 Looper K, Winchester K, Robinson D, *et al.* Best Practices for Chemotherapy Administration in Pediatric Oncology: Quality and Safety Process Improvements (2015). *J Pediatr Oncol Nurs* 2015;**33**:165–72.

68 Low TKL, Tay KH, Fang T, *et al.* Reducing transfers of psychiatric inpatients to emergency rooms of general hospitals in Singapore: a clinical practice improvement project. *Int J Psychiatry Clin Pract* 2017;**21**:50–7.

69 Maqbool T, Raju S, In E. Importance of patient-centred signage and navigation guide in an orthopaedic and plastics clinic. *BMJ Qual Improv reports* 2016;**5**:10.1136/bmjquality.u209473.w3887. eCollection 2016.

70 Margalit BDN, Losi SM, Tishler RB, *et al.* Focus on Quality Quality in Action Ensuring Head and Neck Oncology Patients Receive Recommended Pretreatment Dental Evaluations. *J Clin Oncol* 2015;**32**:no pagination.

71 May-Miller H, Hayter J, Loewenthal L, *et al.* Improving the quality of discharge summaries: implementing updated Academy of Medical Royal Colleges standards at a district general hospital. *BMJ Qual Improv Reports* 2015;**4**:u207268.w2918.

72 McCormack R. Improving the uptake and comprehensiveness of bedside cognitive testing amongst liaison psychiatrists over an eight-month period. *BMJ Qual Improv reports* 2016;**5**:1–6.

73 Mgonja S, Schoening A. Postpartum Depression Screening at Well-Child Appointments: A Quality Improvement Project. *J Pediatr Health Care* Published Online First: 2016.

74 Minhas A, Kyaw TT, Ong BC. Redesigning case selection methods to improve clinical review of inpatient medical records. *Clin Epidemiol Glob Heal* 2015;**3**:66–71.

75 Montella JM, Pelegano JF. Improving the Rate of Colposcopy in an Urban Population of Patients With Known Abnormal Pap Smears. *Am J Med Qual* 2016;**31**:233–9.

76 Mookadam M, Grover M, Pullins C, *et al.* Simple Interventions Improve the Quality of a Missed Lab Appointment Process. *BMJ Qual Improv Reports* 2016;**5**:u205944.w2432.

77 Murphy L, Wells JS, Lachman P, *et al.* A Quality Improvement Initiative in Community Mental Health in the Republic of Ireland. 2015;:1–12.

78 Mustafa A, Mahgoub S. Understanding and overcoming barriers to timely discharge from the pediatric units. *BMJ Qual Improv Reports* 2016;**5**:u209098.w3772.

79 Nelson E, Reynolds P. Inpatient Falls: Improving assessment, documentation, and management. *BMJ Qual Improv reports* 2015;**4**:10.1136/bmjquality.u208575.w3781. eCollection 2015.

80 Nuti A. Improving compliance with iron infusion therapy in the treatment of chronic anemia in haemodialysis patients with chronic kidney disease. *BMJ Qual Improv Reports* 2015;**4**:u204642.w2177.

81 Ozekcin LR, Tuite P, Willner K, *et al.* Simulation education: Early identification of patient physiologic deterioration by acute care nurses. *Clin Nurse Spec* 2015;**29**:166–73.

82 Partridge T, Carluke I, Emmerson K, *et al.* Improving patient reported outcome measures (PROMs) in total knee replacement by changing implant and preserving the infrapatella fatpad: a quality improvement project. *BMJ Qual Improv reports* 2016;**5**:10.1136/bmjquality.u204088.w3767. eCollection 2016.

83 Peh WM, Jia Loh W, chee phua ghee, *et al.* Eliminating guidewire retention during ultrasound guided central venous catheter insertion via an educational program, a modified CVC set, and a drape with reminder stickers. *BMJ Qual Improv Reports* 2016;**5**:u209550.w3941.

84 Prager JD, Ruiz AG, Mooney K, *et al.* Improving operative flow during pediatric airway evaluation a quality-improvement initiative. *JAMA Otolaryngol - Head Neck Surg* 2015;**141**:229–35.

85 O’Reilly A. Improving ward environments and developing skills for discharge with the implementation of self-catering on a low secure forensic unit. *BMJ Qual Improv Reports* 2016;**5**:u210929.w4509.

86 Reynolds M, Jheeta S, Benn J, *et al.* Improving feedback on junior doctors? prescribing errors: Mixed-methods evaluation of a quality improvement project. *BMJ Qual Saf* 2017;**26**:240–7.

87 Riblet NBV, Schlosser EM, Snide JA, *et al.* A clinical care pathway to improve the acute care of patients with glioma. *Neuro-Oncology Pract* 2016;**3**:145–53.

88 Roberts S, Saithna A, Bethune R. Improving theatre efficiency and utilisation through early identification of trauma patients and enhanced communication between teams. *BMJ Qual Improv Reports* 2015;**4**:u206641.w2670-u206641.w2670.

89 Rostami P, Power M, Harrison A, *et al.* Learning from the design, development and implementation of the Medication Safety Thermometer. *Int J Qual Heal Care* 2016;:1–9.

90 Salman M, Subbe C. Alcohol detoxification in Ysbyty Gwynedd: Two small sips or one big gulp? Two-step screening more reliable for identification of alcohol dependency syndrome at risk of delirium tremens for routine care. *BMJ Qual Improv reports* 2015;**4**:10.1136/bmjquality.u206149.w2528. eCollection 2015.

91 6 and Joanne Hilden 2 Jennifer L. Salstrom MD PhD 1 2 3* Rebecca L. Coughlin MEd 1 4 Kathleen Pool MSN CPNP 1 Melissa Bojan BSN 1 Camille Mediavilla BSN 1 William Schwent MBA 4 Michael Rannie MS 5 Dawn Law MBA 5 Michelle Finnerty BS. Prognosis in children with rhabdomyosarcoma: A report of the intergroup rhabdomyosarcoma studies I and II. *J Clin Oncol* 1990;**8**:443–52.

92 Samaan ZM, Brown CM, Morehous J, *et al.* Implementation of a Preventive Services Bundle in Academic Pediatric Primary Care Centers. *Pediatrics* 2016;**137**:e20143136–e20143136.

93 Samji K, Kielar A, Connolly M, *et al.* Quality Improvement Initiative to Increase Consistent Use of Intraluminal Contrast in the Identification of Anastomotic Bowel Leaks on Computed Tomography, Using the Plan-Do-Study-Act Service Approach. *Can Assoc Radiol J* 2017;**68**:4–9.

94 Savarino JR, Kaplan JL, Winter HS, *et al.* Improving Clinical Remission Rates in Pediatric Inflammatory Bowel Disease with Previsit Planning. *BMJ Qual Improv Reports* 2016;**5**:u211063.w4361.

95 Schorr C, Odden A, Evans L, *et al.* Implementation of a multicenter performance improvement program for early detection and treatment of severe sepsis in general medical-surgical wards. *J Hosp Med* 2016;**11 Suppl 1**:S32–9.

96 Shahnazarian V, Karu E, Mehta P. Hepatitis C: improving the quality of screening in a community hospital by implementing an electronic medical record intervention. *BMJ Qual Improv Reports* 2015;**4**:u208549.w3409.

97 Shaikh U, Slee C. Triple Duty: Integrating Graduate Medical Education With Maintenance of Board Certification to Improve Clinician Communication at Hospital Discharge. *J Grad Med Educ* 2015;**7**:462–5.

98 Singh I, Fletcher R, Scanlon L, *et al.* A quality improvement initiative on the management of osteoporosis in older people with Parkinsonism. *BMJ Qual Improv Reports* 2016;**5**:u210921.w5756.

99 Singh I, Okeke J. Reducing inpatient falls in a 100% single room elderly care environment: evaluation of the impact of a systematic nurse training programme on falls risk assessment (FRA). *BMJ Qual Improv Reports* 2016;**5**:u210921.w4741.

100 Stark C, Gent A, Kirkland L. Improving patient flow in pre-operative assessment. *BMJ Qual Improv Reports* 2015;**4**:u201341.w1226.

101 Subramanyam R, Mahmoud M, Buck D, *et al.* Infusion Medication Error Reduction by Two-Person Verification: A Quality Improvement Initiative. *Pediatrics* 2016;**138**:e20154413–e20154413.

102 Sudhanthar S, Turner J, Thakur K, *et al.* Improving viable low cost generic medication prescription rate in primary care pediatric practice. *BMJ Qual Improv reports* 2015;**4**:3–5.

103 Sudhanthar S, Thakur K, Sigal Y, *et al.* Improving validated depression screen among adolescent population in primary care practice using electronic health records (EHR). *BMJ Qual Improv Reports* 2015;**4**:u209517.w3913.

104 Tan RYC, Met-Domestici M, Zhou K, *et al.* Using Quality Improvement Methods and Time-Driven Activity-Based Costing to Improve Value-Based Cancer Care Delivery at a Cancer Genetics Clinic. *J Oncol Pract* 2016;**12**:e320–31.

105 Thomas P, Seale JP, Johnson JA, *et al.* Impact of a Quality Improvement Intervention to Increase Brief Alcohol and Drug Interventions on a Level I Trauma Service. *Am Surg* 2016;**82**:468–73.

106 Thomassen O, Mann C, Mbwana JS, *et al.* Emergency medicine in Zanzibar: the effect of system changes in the emergency department. *Int J Emerg Med* 2015;**8**:22.

107 Titsworth WL, Abram J, Guin P, *et al.* A prospective time-series quality improvement trial of a standardized analgesia protocol to reduce postoperative pain among neurosurgery patients. *J Neurosurg* 2016;**3**:1–10.

108 Tripathi S, Arteaga G, Rohlik G, *et al.* Implementation of Patient-Centered Bedside Rounds in the Pediatric Intensive Care Unit. *J Nurs Care Qual* 2015;**30**:160–6.

109 Ugarte M. Waiting time reduction in intravitreal clinics by optimization of appointment scheduling: balancing demand and supply. *BMJ Qual Improv reports* 2015;**4**:10.1136/bmjquality.u208924.w3618. eCollection 2015.

110 Vetter MJ, Gnp-bc ANP. The Influence of Clinical Decision Support on Diagnostic Accuracy in Nurse Practitioners. 2012;:1–9.

111 Wale M, Kibsey P, Young L, *et al.* New approaches to infection prevention and control: Implementing a risk-based model regionally. *Int J Qual Heal Care* 2016;**28**:405–11.

112 Walton H, Munro W. Improving the quality of handover by addressing handover culture and introducing a new, multi-disciplinary, team-based handover meeting. *BMJ Qual Improv Reports* 2015;**4**:u206069.w2989.

113 Ward J, Spencer R, Soo E, *et al.* Standardising the organisation of clinical equipment on surgical wards at North Bristol NHS Trust: a quality improvement initiative. *BMJ Qual Improv reports* 2015;**4**:10.1136/bmjquality.u208308.w3441. eCollection 2015.

114 Wiemann CM, Hergenroeder AC, Bartley KA, *et al.* Integrating an EMR-based Transition Planning Tool for CYSHCN at a Children’s Hospital: A Quality Improvement Project to Increase Provider Use and Satisfaction. *J Pediatr Nurs* 2015;**30**:776–87.

115 Wittkugel E, Varughese A. Development of a nurse-assisted preanesthesia evaluation program for pediatric outpatient anesthesia. *Paediatr Anaesth* 2015;**25**:719–26.

116 Wrenn G, Kasiah F, Syed I. Using a self-service kiosk to identify behavioural health needs in a primary care clinic serving an urban, underserved population. *J Innov Heal Informatics* 2015;**22**:323–8.

117 Wu S, Tyler A, Logsdon T, *et al.* A Quality Improvement Collaborative to Improve the Discharge Process for Hospitalized Children. *Pediatrics* 2016;**138**:e20143604–e20143604.

118 Yager P, Collins C, Blais C, *et al.* Quality improvement utilizing in-situ simulation for a dual-hospital pediatric code response team. *Int J Pediatr Otorhinolaryngol* 2016;**88**:42–6.

119 Yelland J, Biro MA, Dawson W, *et al.* Bridging the language gap: A co-designed quality improvement project to engage professional interpreters for women duing labour. *Aust Heal Rev* 2017;**41**:499–504.

120 Zuccarelli B, Coffman KA. Reducing after-hours prescription refill requests. *Neurol Clin Pract* 2016;**6**:429–32.
