# Supplementary material for: Can quality improvement improve the quality of care? A systematic review of reported effects and methodological rigor in plan-do-study-act projects
Source: BMC Health Serv Res. 2019 Oct 4;19:683. doi: 10.1186/s12913-019-4482-6 (PMC6778385; doi:10.1186/s12913-019-4482-6)
Supplement: Supplementary file 3 — Projects identified in search that describes PDSA method in sufficient detail to be included for full analysis for framework. (DOCX 145 kb) [file 12913_2019_4482_MOESM3_ESM.docx]

**Additional file 3: Studies identified in search that described PDSA method in sufficient detail to be included for full analysis for framework**

| Author | PDSA thoroughness | Iterative | Type of iterative cycles | Several tests of change in cycle | Small scale testing used | | Scope of QI efforts | Type of scaling in testing | Data over time used | Data over time measurement type | Baseline used | Type of time series | Main type of data used | Theoretical rationale | | Evidence based improvement | Origin of confidence in improvement process |
| --- | --- | --- | --- | --- | --- | --- | --- | --- | --- | --- | --- | --- | --- | --- | --- | --- | --- |
| [1] Afanvi | 3 | Yes | Iterative chain | No | No | Implementation | | N/A | Yes | Regular: Three datapoints or more | Yes | Control Chart | Quantitative | Yes | Yes | | External existing knowledge – Literature (EBM) |
| [2] Ahmad | 3 | Yes | Iterative chain | No | No | Implementation | | N/A | Yes | Regular: Three datapoints or more | Yes | Run Chart | Quantitative | Yes | Yes | | External existing knowledge - Previous QI |
| [3] Ahmed Awaji | 3 | Yes | Iterative chain | No | Yes | Implementation | | Increasing | Yes | Regular: Three datapoints or more | Yes | Run Chart | Quantitative | No | Yes | | External existing knowledge – Literature (EBM) |
| [4] Akinbobuyi | 3 | Yes | Iterative chain | No | No | Implementation | | N/A | No | Irregular: Before and after, or per cycle | Yes | N/A | Quantitative | Yes | Yes | | External existing knowledge – Literature (Guidelines) |
| [5] Alhamid | 3 | Yes | Iterative chain | Yes | No | Implementation | | N/A | Yes | Regular: Three datapoints or more | Yes | Control Chart | Quantitative supplemented | Yes | Yes | | Internally developed knowledge - Logical visualising and description |
| [6] Aljaber | 4 | Yes | Iterative chain | No | No | Testing | | N/A | Yes | Regular: Three datapoints or more | Yes | Run Chart | Quantitative | Yes | Yes | | Internally developed knowledge - Logical visualising and description |
| [7] AlSaleem | 3 | Yes | Iterative chain | Yes | Yes | Testing | | Increasing | Yes | Regular: Three datapoints or more | Yes | Control Chart | Quantitative | Yes | Yes | | Internally developed knowledge - Logical visualising and description |
| [8] Alshieban | 3 | Yes | Iterative chain | Yes | No | Testing | | N/A | Yes | Regular: Three datapoints or more | Yes | Run Chart | Quantitative | Yes | Yes | | Internally developed knowledge - Logical visualising and description |
| [9] Anderson | 3 | Yes | Mix of iterative chains and isolated cycles | No | No | Implementation | | N/A | No | Irregular: Before and after, or per cycle | Yes | N/A | Quantitative | No | Yes | | Internally developed knowledge - Logical visualising and description |
| [10] Andersson | 4 | Yes | Iterative chain | No | No | Testing | | N/A | No | Irregular: Before and after, or per cycle | Yes | N/A | Quantitative | Yes | Yes | | External existing knowledge – Literature (EBM) |
| [11] Aragona | 3 | Yes | Mix of iterative chains and isolated cycles | No | No | Testing | | N/A | Yes | Regular: Three datapoints or more | Yes | Control Chart | Quantitative supplemented | Yes | Yes | | Combination of external and internal |
| [12] Aung | 3 | Yes | Iterative chain | No | No | Testing | | N/A | No | Irregular: Before and after, or per cycle | Yes | N/A | Quantitative | No | Yes | | Not stated |
| [13] Bailey | 3 | Yes | Multiple (isolated) iterative chains | No | No | Implementation | | N/A | Yes | Regular: Three datapoints or more | Yes | Control Chart | Quantitative | No | Yes | | External existing knowledge - Previous QI |
| [14] Bays | 3 | Yes | Iterative chain | No | No | Implementation | | N/A | Yes | Regular: Three datapoints or more | Yes | Control Chart | Quantitative | No | Yes | | Internally developed knowledge - Logical visualising and description |
| [15] Bell | 3 | No | Multiple isolated cycles | No | No | Implementation | | N/A | Yes | Regular: Three datapoints or more | Yes | Run Chart | Quantitative supplemented | No | Yes | | External existing knowledge – Literature (EBM) |
| [16] Bock | 3 | No | Multiple isolated cycles | No | No | Implementation | | N/A | Yes | Regular: Three datapoints or more | Yes | Control Chart | Quantitative | No | Yes | | Not stated |
| [17] Bray | 3 | Yes | Iterative chain | Yes | No | Testing and implementing | | N/A | No | Irregular: Before and after, or per cycle | Yes | N/A | Quantitative | No | Yes | | Not stated |
| [18] Brown | 3 | No | Multiple isolated cycles | No | No | Testing | | N/A | Yes | Regular: Three datapoints or more | Yes | Control Chart | Quantitative | No | Yes | | Internally developed knowledge - Logical visualising and description |
| [19] Burchett | 3 | Yes | Multiple (isolated) iterative chains | No | Yes | Testing and implementing | | Increasing | Yes | Regular: Three datapoints or more | Yes | Control Chart | Quantitative | No | Yes | | Not stated |
| [20] Chartier | 3 | Yes | Multiple (isolated) iterative chains | No | No | Testing | | N/A | Yes | Regular: Three datapoints or more | Yes | Control Chart | Quantitative | Yes | Yes | | Internally developed knowledge - Logical visualising and description |
| [21] Cohen | 3 | Yes | Mix of iterative chains and isolated cycles | Yes | No | Testing | | N/A | Yes | Regular: Three datapoints or more | Yes | Control Chart | Quantitative | No | Yes | | Internally developed knowledge - Logical visualising and description |
| [22] Cooper | 3 | Yes | Iterative chain | No | No | Testing | | N/A | No | Irregular: Before and after, or per cycle | Yes | N/A | Quantitative | No | Yes | | External existing knowledge – Literature (Guidelines) |
| [23] Cottney | 3 | Yes | Iterative chain | No | No | Implementation | | N/A | Yes | Regular: Three datapoints or more | Yes | Control Chart | Quantitative | Yes | Yes | | External existing knowledge - Previous QI |
| [24] Croxford | 4 | Yes | Iterative chain | No | No | Implementation | | N/A | Yes | Regular: Three datapoints or more | Yes | Run Chart | Quantitative | Yes | Yes | | Internally developed knowledge - Logical visualising and description |
| [25] Curatolo | 3 | Yes | Iterative chain | No | No | Implementation | | N/A | No | Irregular: Before and after, or per cycle | No | N/A | Quantitative | Yes | Yes | | Not stated |
| [26] Donnelly | 3 | Yes | Mix of iterative chains and isolated cycles | No | No | Testing and implementing | | N/A | Yes | Regular: Three datapoints or more | Yes | Run Chart | Quantitative supplemented | No | Not stated | | Internally developed knowledge - Logical visualising and description |
| [27] Dunbar | 3 | No | Multiple isolated cycles | No | No | Testing | | N/A | Yes | Regular: Three datapoints or more | Yes | Run Chart | Quantitative | No | Yes | | Internally developed knowledge - Logical visualising and description |
| [28] Dykes | 3 | Yes | Multiple (isolated) iterative chains | No | No | Implementation | | N/A | Yes | Regular: Three datapoints or more | Yes | Control Chart | Quantitative supplemented | No | Yes | | External existing knowledge – Literature (EBM) |
| [29] Fontanez-Nieves | 3 | Yes | Iterative chain | No | No | Implementation | | N/A | Yes | Regular: Three datapoints or more | Yes | Control Chart | Quantitative | Yes | Yes | | External existing knowledge – Literature (EBM) |
| [30] Frost | 4 | No | Multiple isolated cycles | No | No | Implementation | | N/A | Yes | Regular: Three datapoints or more | Yes | Run Chart | Quantitative | Yes | Yes | | Internally developed knowledge - Logical visualising and description |
| [31] Goulding | 3 | Yes | Iterative chain | Yes | No | Implementation | | N/A | No | Irregular: Before and after, or per cycle | Yes | N/A | Quantitative supplemented | Yes | Yes | | Combination of external and internal |
| [32] Guo | 3 | Yes | Iterative chain | No | No | Testing | | N/A | Yes | Regular: Three datapoints or more | Yes | Run Chart | Quantitative | No | Yes | | Combination of external and internal |
| [33] Hale | 3 | Yes | Iterative chain | No | No | Implementation | | N/A | Yes | Regular: Three datapoints or more | Yes | Run Chart | Quantitative | No | Yes | | Internally developed knowledge - Logical visualising and description |
| [34] Hall | 3 | Yes | Iterative chain | No | No | Testing | | N/A | Yes | Regular: Three datapoints or more | Yes | Run Chart | Quantitative | No | Yes | | Internally developed knowledge - Logical visualising and description |
| [35] Hanison | 3 | No | Multiple isolated cycles | Yes | No | Testing | | N/A | No | Irregular: Before and after, or per cycle | Yes | N/A | Quantitative | No | Yes | | Not stated |
| [36] Hatoun | 3 | Yes | Iterative chain | Yes | No | Testing | | N/A | Yes | Regular: Three datapoints or more | Yes | Control Chart | Quantitative | Yes | Yes | | Internally developed knowledge - Logical visualising and description |
| [37] Hayes | 3 | No | Multiple isolated cycles | Yes | No | Testing | | N/A | No | Irregular: Before and after, or per cycle | Yes | N/A | Quantitative | Yes | Yes | | Internally developed knowledge - Logical visualising and description |
| [38] Hendricks | 3 | Yes | Iterative chain | No | No | Testing | | N/A | Yes | Regular: Three datapoints or more | Yes | Run Chart | Quantitative supplemented | No | Yes | | Combination of external and internal |
| [39] Holmes | 3 | Yes | Mix of iterative chains and isolated cycles | No | Yes | Testing and implementing | | Unclear | Yes | Regular: Three datapoints or more | Yes | Control Chart | Quantitative supplemented | Yes | Yes | | Combination of external and internal |
| [40] Jones | 3 | No | Multiple isolated cycles | Yes | No | Implementation | | N/A | Yes | Regular: Three datapoints or more | Yes | Run Chart | Quantitative | No | Yes | | Combination of external and internal |
| [41]Kulik | 4 | Yes | Iterative chain | No | No | Testing | | N/A | Yes | Regular: Three datapoints or more | Yes | Run Chart | Quantitative supplemented | Yes | Yes | | Combination of external and internal |
| [42] Li | 4 | No | Yes | Yes | No | Testing | | N/A | No | A single data point, after cycle | Yes | N/A | Quantitative | No | Yes | | Combination of external and internal |
| [43] Looper | 3 | Yes | Iterative chain | Yes | Yes | Testing | | Non-increasing | Yes | Regular: Three datapoints or more | No | Run Chart | Quantitative | No | Yes | | Not stated |
| [44] Maqbool | 3 | No | Multiple isolated cycles | No | No | Testing | | N/A | No | Irregular: Before and after, or per cycle | Yes | N/A | Quantitative supplemented | No | Yes | | Not stated |
| [45] May-Miller | 3 | Yes | Iterative chain | No | No | Testing | | N/A | Yes | Regular: Three datapoints or more | Yes | Run Chart | Quantitative | No | Yes | | External existing knowledge – Literature (EBM) |
| [46] McCormack | 3 | No | Multiple isolated cycles | No | No | Testing | | N/A | No | Irregular: Before and after, or per cycle | Yes | N/A | Quantitative | No | Yes | | Not stated |
| [47] Mgonja | 3 | No | Multiple isolated cycles | No | No | Implementation | | N/A | Yes | Regular: Three datapoints or more | No | Not stated | Quantitative | No | Yes | | External existing knowledge – Literature (EBM) |
| [48] Minhas | 3 | Yes | Iterative chain | No | No | Testing and implementing | | N/A | No | Irregular: Before and after, or per cycle | Yes | Run Chart | Quantitative supplemented | No | Not stated | | Not stated |
| [49] Mookadam | 3 | Yes | Iterative chain | No | No | Testing | | N/A | No | Irregular: Before and after, or per cycle | Yes | N/A | Quantitative | No | Yes | | Not stated |
| [50] Murphy | 3 | Yes | Mix of iterative chains and isolated cycles | Yes | Yes | Testing and implementing | | Non-increasing | Yes | Regular: Three datapoints or more | No | Control Chart | Quantitative & Qualitative used to inform cycles | No | Yes | | External existing knowledge – Literature (EBM) |
| [51] Mustafa | 4 | Yes | Iterative chain | Yes | No | Testing | | N/A | Yes | Regular: Three datapoints or more | Yes | Run Chart | Quantitative | Yes | Yes | | Internally developed knowledge - Logical visualising and description |
| [52] Nelson | 3 | Yes | Iterative chain | No | No | Implementation | | N/A | No | Irregular: Before and after, or per cycle | Yes | N/A | Quantitative | No | Yes | | External existing knowledge - Benchmarking |
| [53] Nuti | 4 | Yes | Iterative chain | No | No | Implementation | | N/A | No | No quantitative data reported | No | N/A | Quantitative but not presented | No | Not stated | | Not stated |
| [54] Ozekcin | 4 | No | Yes | Yes | No | Implementation | | N/A | No | A single data point, after cycle | Yes | N/A | Quantitative & Qualitative used to inform cycles | Yes | Yes | | Combination of external and internal |
| [55] Partridge | 3 | No | Multiple isolated cycles | No | No | Implementation | | N/A | No | Irregular: Before and after, or per cycle | Yes | N/A | Quantitative | No | Yes | | External existing knowledge – Literature (EBM) |
| [56] Peh | 3 | Yes | Iterative chain | No | No | Testing | | N/A | No | A single data point, after cycle | Yes | N/A | Quantitative supplemented | Yes | Yes | | Internally developed knowledge - Logical visualising and description |
| [57] Reilly | 3 | Yes | Iterative chain | No | No | Testing and implementing | | N/A | Yes | Regular: Three datapoints or more | No | Control Chart | Quantitative supplemented | No | Yes | | External existing knowledge - Benchmarking |
| [58] Roberts | 4 | Yes | Iterative chain | No | No | Implementation | | N/A | Yes | Regular: Three datapoints or more | Yes | Control Chart | Quantitative | Yes | Yes | | External existing knowledge - Previous QI |
| [59] Rostami | 4 | Yes | Iterative chain | Yes | Yes | Testing, implementing and spreading | | Increasing | Yes | Regular: Three datapoints or more | Yes | Run Chart | Quantitative | Yes | Yes | | Combination of external and internal |
| [60] Salman | 3 | Yes | Iterative chain | No | Yes | Testing and implementing | | Increasing | No | A single data point, after cycle | Yes | N/A | Quantitative | No | Yes | | External existing knowledge – Literature (Guidelines) |
| [61] Salstrom | 3 | Yes | Mix of iterative chains and isolated cycles | No | No | Testing and implementing | | N/A | Yes | Regular: Three datapoints or more | Yes | Control Chart | Quantitative | No | Yes | | Internally developed knowledge - Logical visualising and description |
| [62] Samji | 3 | Yes | Iterative chain | No | No | Implementation | | N/A | No | Irregular: Before and after, or per cycle | Yes | N/A | Quantitative | No | Yes | | External existing knowledge – Literature (EBM) |
| [63] Savarino | 3 | Yes | Mix of iterative chains and isolated cycles | No | Yes | Implementation | | Increasing | Yes | Regular: Three datapoints or more | Yes | Control Chart | Quantitative supplemented | No | Yes | | External existing knowledge - Previous QI |
| [64] Singh | 3 | Yes | Mix of iterative chains and isolated cycles | Yes | No | Implementation | | N/A | Yes | Regular: Three datapoints or more | Yes | Run Chart | Quantitative | Yes | Yes | | External existing knowledge – Literature (Guidelines) |
| [65] Sudhanthar | 3 | Yes | Mix of iterative chains and isolated cycles | No | No | Testing | | N/A | Yes | Regular: Three datapoints or more | Yes | Run Chart | Quantitative | No | Yes | | Not stated |
| [66] Sudhanthar | 3 | Yes | Iterative chain | No | No | Implementation | | N/A | Yes | Regular: Three datapoints or more | Yes | Run Chart | Quantitative | No | Yes | | External existing knowledge – Literature (Guidelines) |
| [67] Tan | 3 | No | Multiple isolated cycles | No | No | Testing | | N/A | Yes | Regular: Three datapoints or more | Yes | Control Chart | Quantitative | No | Yes | | Internally developed knowledge - Logical visualising and description |
| [68] Thomassen | 3 | Yes | Mix of iterative chains and isolated cycles | No | No | Testing | | N/A | Yes | Regular: Three datapoints or more | Yes | Control Chart | Quantitative | No | Not stated | | Not stated |
| [69] Tripathi | 4 | Yes | Iterative chain | No | No | Implementation | | N/A | No | Irregular: Before and after, or per cycle | Yes | N/A | Quantitative supplemented | No | Yes | | External existing knowledge – Literature (EBM) |
| [70] Walton | 3 | Yes | Iterative chain | No | No | Implementation | | N/A | Yes | Regular: Three datapoints or more | Yes | Control Chart | Quantitative & Qualitative used to inform cycles | No | Yes | | Not stated |
| [71] Ward | 3 | Yes | Iterative chain | No | Yes | Implementation | | Increasing | No | A single data point, after cycle | Yes | N/A | Quantitative | No | Yes | | External existing knowledge - Benchmarking |
| [72] Wiemann | 3 | Yes | Mix of iterative chains and isolated cycles | No | No | Implementation | | N/A | Yes | Regular: Three datapoints or more | No | Run Chart | Quantitative supplemented | No | Yes | | External existing knowledge – Literature (EBM) |

| **Elaboration table** | |
| --- | --- |
| **PDSA thoroughness** | **Main type of data used** |
| ***1 or 2 = insufficient documentation of PDSA***  1 = No details of cycles  2 = Themes of cycles but no additional details  **Studies with 1 or 2 were excluded from full analysis.**  ***3 or 4 = sufficient documentation of PDSA***  3 = details of individual cycles but not stages of cycles  4 = details of cycles including separate information on stages of cycles | Quantitative supplemented = The quantitative data was supplemented by measures on patient/staff satisfaction/experience  Quantitative & Qualitative = Quantitative & Qualitative was used to inform cycles |

1 Afanvi KA. From many deaths to some few cases of drug-resistant tuberculosis: travelling with the systems quality improvement model in Lacs Health District, Togo. *BMJ Qual Improv Reports* 2015;**4**:u201413.w1473.

2 Ahmad AN, Leyla Byrne M, Imambaccus N, *et al.* Venous thromboembolism capture on electronic systems in obstetrics patients at St Thomas’ Hospital. *BMJ Qual Improv Reports* 2016;**5**:u212405.w5122.

3 Ahmed Awaji M, Al-Surimi K. Promoting the role of patients in improving hand hygiene compliance amongst health care workers. *BMJ Qual Improv Reports* 2016;**5**:u210787.w4336.

4 Akinbobuyi O, shalders louise, Nokes T. Ensuring timely thromboprophylaxis on a Medical Assessment Unit. *BMJ Qual Improv Reports* 2016;**5**:u212414.w4934.

5 Alhamid SM, Lee DX-Y, Wong HM, *et al.* Implementing electronic handover: interventions to improve efficiency, safety and sustainability. *Int J Qual Heal Care* 2016;**28**:608–14.

6 Aljaber A, Al-Surimi K. Promoting oral health practice among patients with diabetes attending primary health care clinics. *BMJ Qual Improv Reports* 2015;**4**:u209172.w3737.

7 Al Saleem N, Al-Surimi K. Reducing the occurrence of errors in a laboratory’s specimen receiving and processing department. *BMJ Qual Improv Reports* 2016;**5**:u211474.w4624.

8 Alshieban S, Al-Surimi K. Reducing turnaround time of surgical pathology reports in pathology and laboratory medicine departments. *BMJ Qual Improv reports* 2015;**4**:u209223.w3773.

9 Anderson C, McNab D. Quality improvement project using a care bundle approach on the management of the immediate discharge document (IDD) within a single general practice. *BMJ Qual Improv reports* 2015;**4**:10.1136/bmjquality.u204819.w3337. eCollection 2015.

10 Andersson J, Bull T, Paul D, *et al.* Safer fluid prescribing at North Bristol Trust: Bringing practice in line with NICE Guidance with a redesign of the fluid prescription chart. *BMJ Qual Improv Reports* 2015;**4**:u203816.w1911.

11 Aragona E, Ponce-Rios J, Garg P, *et al.* A Quality Improvement Project to Increase Nurse Attendance on Pediatric Family Centered Rounds. *J Pediatr Nurs* 2016;**31**:e3–9.

12 Aung TH, Judith Beck A, Siese T, *et al.* Less is more: a project to reduce the number of PIMs (potentially inappropriate medications) on an elderly care ward. *BMJ Qual Improv reports* 2016;**5**:1–4.

13 Bailey J, Page B, Ndimande N, *et al.* Absconding: reducing failure to return in adult mental health wards. *BMJ Qual Improv Reports* 2016;**5**:u209837.w5117.

14 Bays A, Wahl E, Daikh DI, *et al.* Implementation of disease activity measurement for rheumatoid arthritis patients in an academic rheumatology clinic. *BMC Health Serv Res* 2016;**16**:384.

15 Bell A, Gallacher N. Succeeding in Sustained Reduction in the use of Restraint using the Improvement Model. *BMJ Qual Improv Reports* 2016;**5**:u211050.w4430.

16 Bock A, Chintamaneni K, Rein L, *et al.* Improving pneumococcal vaccination rates of medical inpatients in urban Nepal using quality improvement measures. *BMJ Qual Improv Reports* 2016;**5**:u212047.w4835.

17 Bray L. Improving cranial ultrasound scanning strategy in neonates. *BMJ Qual Improv Reports* 2016;**5**:u210346.w4219.

18 Brown J, Fawzi W, Shah A, *et al.* Low stimulus environments: reducing noise levels in continuing care. *BMJ Qual Improv Reports* 2016;**5**:u207447.w4214.

19 Burchett P, Harpin S, Petersen-Smith A, *et al.* Improving a Urine Culture Callback Follow-up System in a Pediatric Emergency Department. *J Pediatr Heal Care* 2015;**29**:518–25.

20 Chartier LB, Simoes L, Kuipers M, *et al.* Improving Emergency Department flow through optimized bed utilization. *BMJ Qual Improv* 2016;**5**:1–5.

21 Cohen ES, Ogrinc G, Taylor T, *et al.* Influenza vaccination rates for hospitalised patients: A multiyear quality improvement effort. *BMJ Qual Saf* 2015;**24**:221–7.

22 Cooper D. Improving the rate of Patient Feedback for a Later Life Mental Health Liaison Team. *BMJ Qual Improv Reports* 2016;**5**:u210384.w4457.

23 Cottney A. Using league tables to reduce missed dose medication errors on mental healthcare of older people wards. *BMJ Qual Improv Reports* 2015;**4**:u204237.w3567.

24 Croxford A, Clare A, McCurdy K. Introduction of a Venous Thromboembolism Prophylaxis Protocol for Older Adult Psychiatric Patients. *BMJ Qual Improv Reports* 2015;**4**:u205852.w3226.

25 Curatolo N, Gutermann L, Devaquet N, *et al.* Reducing medication errors at admission: 3 cycles to implement, improve and sustain medication reconciliation. *Int J Clin Pharm* 2014;**37**:113–20.

26 Donnelly P. Improving reporting of critical incidents through education and involvement. *BMJ Qual Improv Reports* 2015;**4**:u206996.w3776.

27 Dunbar J, George J. Mortality meetings in geriatric medicine: strategies for improvement. *BMJ Qual Improv Reports* 2015;**4**:u202625.w3247.

28 Dykes D, Williams E, Margolis P, *et al.* Improving pediatric Inflammatory Bowel Disease (IBD) follow-up. *BMJ Qual Improv Reports* 2016;**5**:u208961.w3675.

29 Fontánez-Nieves TD, Frost M, Anday E, *et al.* Prevention of unplanned extubations in neonates through process standardization. *J Perinatol* 2016;**36**:469–73.

30 Frost L. Reducing the overuse of βhCG measurements in the emergency gynaecology clinic. *BMJ Qual Improv Reports* 2016;**5**:u210039.w4218.

31 Goulding L, Parke H, Maharaj R, *et al.* Improving critical care discharge summaries: a collaborative quality improvement project using PDSA. *BMJ Qual Improv Reports* 2015;**4**:u203938.w3268.

32 Guo M, Bosnyak S, Bontempo T, *et al.* Let’s Talk About Sex! - Improving sexual health for patients in stroke rehabilitation. *BMJ Qual Improv Reports* 2015;**4**:u207288.w2926.

33 Hale G, McNab D. Developing a ward round checklist to improve patient safety. *BMJ Qual Improv Reports* 2015;**4**:u204775.w2440-u204775.w2440.

34 Hall W. Improving the safety of prescriptions of domperidone in primary care: implementing MHRA advice. *BMJ Qual Improv Reports* 2016;**5**:u209711.w4039.

35 Hanison J, Conway D. A multifaceted approach to prevention of delirium on intensive care. *BMJ Qual Improv Reports* 2015;**4**:u209656.w4000.

36 Hatoun J, Bair-Merritt M, Cabral H, *et al.* Increasing Medication Possession at Discharge for Patients With Asthma: The Meds-in-Hand Project. *Pediatrics* 2016;**137**:e20150461–e20150461.

37 Hayes RM, Wickline A, Hensley C, *et al.* A Quality Improvement Project to Improve Family Recognition of Medical Team Member Roles. *Hosp Pediatr* 2015;**5**:480–6.

38 Hendrickson CD, Saini S, Pothuloori A, *et al.* Assessing Referrals and Improving Information Availability for Consultations in an Academic Endocrinology Clinic. Endocr. Pract. 2017;**23**:190–8.

39 Holmes A V., Atwood EC, Whalen B, *et al.* Rooming-In to Treat Neonatal Abstinence Syndrome: Improved Family-Centered Care at Lower Cost. *Pediatrics* 2016;**137**:e20152929–e20152929.

40 Jones CM, Stewart C, Roszell SS. Beyond best practice implementing a unit-based CLABSI project. *J Nurs Care Qual* 2015;**30**:24–30.

41 Kulik W, Shah A. Role of peer support workers in improving patient experience in Tower Hamlets Specialist Addiction Unit. *BMJ Qual Improv Reports* 2016;**5**:u205967.w2458.

42 Li CH, Traube LE, Lu DS, *et al.* Implementation and Results of a Percutaneous Renal Allograft Biopsy Protocol to Reduce Complication Rate. *J Am …* 2016;**13**:549–53.

43 Looper K, Winchester K, Robinson D, *et al.* Best Practices for Chemotherapy Administration in Pediatric Oncology: Quality and Safety Process Improvements (2015). *J Pediatr Oncol Nurs* 2015;**33**:165–72.

44 Maqbool T, Raju S, In E. Importance of patient-centred signage and navigation guide in an orthopaedic and plastics clinic. *BMJ Qual Improv reports* 2016;**5**:10.1136/bmjquality.u209473.w3887. eCollection 2016.

45 May-Miller H, Hayter J, Loewenthal L, *et al.* Improving the quality of discharge summaries: implementing updated Academy of Medical Royal Colleges standards at a district general hospital. *BMJ Qual Improv Reports* 2015;**4**:u207268.w2918.

46 McCormack R. Improving the uptake and comprehensiveness of bedside cognitive testing amongst liaison psychiatrists over an eight-month period. *BMJ Qual Improv reports* 2016;**5**:1–6.

47 Mgonja S, Schoening A. Postpartum Depression Screening at Well-Child Appointments: A Quality Improvement Project. *J Pediatr Health Care* Published Online First: 2016.

48 Minhas A, Kyaw TT, Ong BC. Redesigning case selection methods to improve clinical review of inpatient medical records. *Clin Epidemiol Glob Heal* 2015;**3**:66–71.

49 Mookadam M, Grover M, Pullins C, *et al.* Simple Interventions Improve the Quality of a Missed Lab Appointment Process. *BMJ Qual Improv Reports* 2016;**5**:u205944.w2432.

50 Murphy L, Wells JS, Lachman P, *et al.* A Quality Improvement Initiative in Community Mental Health in the Republic of Ireland. 2015;:1–12.

51 Mustafa A, Mahgoub S. Understanding and overcoming barriers to timely discharge from the pediatric units. *BMJ Qual Improv Reports* 2016;**5**:u209098.w3772.

52 Nelson E, Reynolds P. Inpatient Falls: Improving assessment, documentation, and management. *BMJ Qual Improv reports* 2015;**4**:10.1136/bmjquality.u208575.w3781. eCollection 2015.

53 Nuti A. Improving compliance with iron infusion therapy in the treatment of chronic anemia in haemodialysis patients with chronic kidney disease. *BMJ Qual Improv Reports* 2015;**4**:u204642.w2177.

54 Ozekcin LR, Tuite P, Willner K, *et al.* Simulation education: Early identification of patient physiologic deterioration by acute care nurses. *Clin Nurse Spec* 2015;**29**:166–73.

55 Partridge T, Carluke I, Emmerson K, *et al.* Improving patient reported outcome measures (PROMs) in total knee replacement by changing implant and preserving the infrapatella fatpad: a quality improvement project. *BMJ Qual Improv reports* 2016;**5**:10.1136/bmjquality.u204088.w3767. eCollection 2016.

56 Peh WM, Jia Loh W, chee phua ghee, *et al.* Eliminating guidewire retention during ultrasound guided central venous catheter insertion via an educational program, a modified CVC set, and a drape with reminder stickers. *BMJ Qual Improv Reports* 2016;**5**:u209550.w3941.

57 O’Reilly A. Improving ward environments and developing skills for discharge with the implementation of self-catering on a low secure forensic unit. *BMJ Qual Improv Reports* 2016;**5**:u210929.w4509.

58 Roberts S, Saithna A, Bethune R. Improving theatre efficiency and utilisation through early identification of trauma patients and enhanced communication between teams. *BMJ Qual Improv Reports* 2015;**4**:u206641.w2670-u206641.w2670.

59 Rostami P, Power M, Harrison A, *et al.* Learning from the design, development and implementation of the Medication Safety Thermometer. *Int J Qual Heal Care* 2016;:1–9.

60 Salman M, Subbe C. Alcohol detoxification in Ysbyty Gwynedd: Two small sips or one big gulp? Two-step screening more reliable for identification of alcohol dependency syndrome at risk of delirium tremens for routine care. *BMJ Qual Improv reports* 2015;**4**:10.1136/bmjquality.u206149.w2528. eCollection 2015.

61 6 and Joanne Hilden 2 Jennifer L. Salstrom MD PhD 1 2 3* Rebecca L. Coughlin MEd 1 4 Kathleen Pool MSN CPNP 1 Melissa Bojan BSN 1 Camille Mediavilla BSN 1 William Schwent MBA 4 Michael Rannie MS 5 Dawn Law MBA 5 Michelle Finnerty BS. Prognosis in children with rhabdomyosarcoma: A report of the intergroup rhabdomyosarcoma studies I and II. *J Clin Oncol* 1990;**8**:443–52.

62 Samji K, Kielar A, Connolly M, *et al.* Quality Improvement Initiative to Increase Consistent Use of Intraluminal Contrast in the Identification of Anastomotic Bowel Leaks on Computed Tomography, Using the Plan-Do-Study-Act Service Approach. *Can Assoc Radiol J* 2017;**68**:4–9.

63 Savarino JR, Kaplan JL, Winter HS, *et al.* Improving Clinical Remission Rates in Pediatric Inflammatory Bowel Disease with Previsit Planning. *BMJ Qual Improv Reports* 2016;**5**:u211063.w4361.

64 Singh I, Fletcher R, Scanlon L, *et al.* A quality improvement initiative on the management of osteoporosis in older people with Parkinsonism. *BMJ Qual Improv Reports* 2016;**5**:u210921.w5756.

65 Sudhanthar S, Turner J, Thakur K, *et al.* Improving viable low cost generic medication prescription rate in primary care pediatric practice. *BMJ Qual Improv reports* 2015;**4**:3–5.

66 Sudhanthar S, Thakur K, Sigal Y, *et al.* Improving validated depression screen among adolescent population in primary care practice using electronic health records (EHR). *BMJ Qual Improv Reports* 2015;**4**:u209517.w3913.

67 Tan RYC, Met-Domestici M, Zhou K, *et al.* Using Quality Improvement Methods and Time-Driven Activity-Based Costing to Improve Value-Based Cancer Care Delivery at a Cancer Genetics Clinic. *J Oncol Pract* 2016;**12**:e320–31.

68 Thomassen O, Mann C, Mbwana JS, *et al.* Emergency medicine in Zanzibar: the effect of system changes in the emergency department. *Int J Emerg Med* 2015;**8**:22.

69 Tripathi S, Arteaga G, Rohlik G, *et al.* Implementation of Patient-Centered Bedside Rounds in the Pediatric Intensive Care Unit. *J Nurs Care Qual* 2015;**30**:160–6.

70 Walton H, Munro W. Improving the quality of handover by addressing handover culture and introducing a new, multi-disciplinary, team-based handover meeting. *BMJ Qual Improv Reports* 2015;**4**:u206069.w2989.

71 Ward J, Spencer R, Soo E, *et al.* Standardising the organisation of clinical equipment on surgical wards at North Bristol NHS Trust: a quality improvement initiative. *BMJ Qual Improv reports* 2015;**4**:10.1136/bmjquality.u208308.w3441. eCollection 2015.

72 Wiemann CM, Hergenroeder AC, Bartley KA, *et al.* Integrating an EMR-based Transition Planning Tool for CYSHCN at a Children’s Hospital: A Quality Improvement Project to Increase Provider Use and Satisfaction. *J Pediatr Nurs* 2015;**30**:776–87.
